# Heterogeneity and longevity of antibody memory to viruses and vaccines

**DOI:** 10.1371/journal.pbio.2006601

**Published:** 2018-08-10

**Authors:** Alice Antia, Hasan Ahmed, Andreas Handel, Nichole E. Carlson, Ian J. Amanna, Rustom Antia, Mark Slifka

**Affiliations:** 1 Carleton College, Northfield, Minnesota, United States of America; 2 Department of Biology, Emory University, Atlanta, Georgia, United States of America; 3 Department of Epidemiology and Biostatistics, University of Georgia, Athens, United States of America; 4 Department of Biostatistics and Informatics, University of Colorado Anschutz Medical Campus, Colorado, United States of America; 5 Najít Technologies, Inc., Beaverton, Oregon, United States of America; 6 Division of Neuroscience, Oregon National Primate Research Center, Beaverton, Oregon, United States of America; Weatherall Institute of Molecular Medicine, University of Oxford, United States of America

## Abstract

Determining the duration of protective immunity requires quantifying the magnitude and rate of loss of antibodies to different virus and vaccine antigens. A key complication is heterogeneity in both the magnitude and decay rate of responses of different individuals to a given vaccine, as well as of a given individual to different vaccines. We analyzed longitudinal data on antibody titers in 45 individuals to characterize the extent of this heterogeneity and used models to determine how it affected the longevity of protective immunity to measles, rubella, vaccinia, tetanus, and diphtheria. Our analysis showed that the magnitude of responses in different individuals varied between 12- and 200-fold (95% coverage) depending on the antigen. Heterogeneity in the magnitude and decay rate contribute comparably to variation in the longevity of protective immunity between different individuals. We found that some individuals have, on average, slightly longer-lasting memory than others—on average, they have higher antibody levels with slower decay rates. We identified different patterns for the loss of protective levels of antibodies to different vaccine and virus antigens. Specifically, we found that for the first 25 to 50 years, virtually all individuals have protective antibody titers against diphtheria and tetanus, respectively, but about 10% of the population subsequently lose protective immunity per decade. In contrast, at the outset, not all individuals had protective titers against measles, rubella, and vaccinia. However, these antibody titers wane much more slowly, with a loss of protective immunity in only 1% to 3% of the population per decade. Our results highlight the importance of long-term longitudinal studies for estimating the duration of protective immunity and suggest both how vaccines might be improved and how boosting schedules might be reevaluated.

## Author summary

Immunological memory, mediated by antibodies, is a hallmark of immunity. A key problem for determining the longevity of protective immunity is heterogeneity in the responses of different individuals. We characterize the extent of this heterogeneity and determine how it affects the longevity of protection. We found that some individuals have higher antibody titers and these same individuals tend to have slower decay rates than others. We also found substantial heterogeneity in both the magnitude and decay rate of responses. Furthermore, differences in these two factors contribute comparably to the variation in antibody titers between different individuals over their lifetime. We then use statistical models to determine how variation in the magnitude and decay rate affect how protective immunity is lost at the population level to different virus and vaccine antigens. We identified different patterns for the loss of protective immunity elicited by protein immunization (tetanus and diphtheria) versus replicating viruses (measles, rubella, and vaccinia). While our results agree with the conventional view that antibodies elicited by protein immunization decay faster than those elicited by replicating viruses, we found that this is compensated for by the higher magnitude of responses (relative to the level for protection) for tetanus and diphtheria. Indeed, for the first 4 decades, a higher fraction of vaccinated individuals have protective immunity to tetanus and diphtheria than to measles, rubella, and vaccinia.

## Introduction

Immune memory is a cardinal feature of the adaptive immune response of vertebrates and is the principle that underlies vaccination [1–3]. Immunological memory arises as a consequence of the increase in the magnitude of the antigen-specific response, augmented by increases in the quality of the response [1,4].

A major problem in quantifying the duration of immune memory in humans is the long timescale involved: immunity typically lasts for many decades [3,5–10]. One approach is to undertake cross-sectional studies that measure immunity in individuals at different times following immunization [5,7,8,11]. While cross-sectional studies provide an estimate of the average rate of loss of immunity, it is difficult to determine the variation in the rate of loss of immunity among different individuals. There are only a few longitudinal studies that follow the decline in immunity to a vaccine and/or virus antigen over time in different individuals [12–15]. Most of these studies focus on the responses to a single vaccine or virus. In this analysis, we used data from a longitudinal study that followed the antibody levels to a panel of 7 vaccine or virus antigens in serum samples drawn from 45 individuals over several decades, as described in detail in ref [12]. The first paper describing this dataset found that antibody responses to virus infections or vaccination with live-attenuated viruses (vaccinia, measles, mumps, rubella, varicella zoster virus [VZV]) were remarkably stable, with half-lives ranging from 50 to over 200 years. In contrast, the antibodies elicited by protein antigens (tetanus and diphtheria toxoids) waned more rapidly, with half-lives of 11 and 19 years, respectively.

In the current study, we asked two sets of questions. The first was to quantify the heterogeneity in antibody responses to different vaccines and viruses at the population level. We wanted to determine the extent to which this heterogeneity depended on the vaccine or virus, the individual (e.g., strong versus weak responders), and the interactions between the vaccine or virus and the individual. A second goal was to use these data to quantify the time to loss of protective immunity to each vaccine or virus at the population level. In particular, we wanted to know both the average duration of protective immunity to each vaccine or virus antigen as well as the extent of the variation of the time to loss of protection between different individuals in the population.

**Fig 1:**
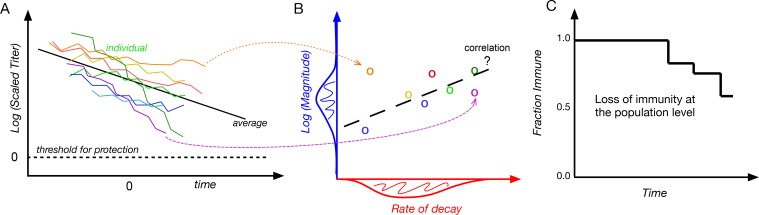
Schematic of the data and analysis. Panel A: antibody titers in different individuals (different colors) to a given vaccine. The titer is scaled to the threshold of protection (dotted line) and shifted so the mean timepoint for each individual is at time = 0. Panels B and C illustrate some of the questions we addressed. Panel B shows a plot of the magnitude of the response and its rate of decay in different individuals. The plot also shows the extent of variation in the magnitude (shaded blue), the rate of decay (shaded red), and a possible correlation between them (dashed black). Panel C shows a plot of how protective immunity is lost at the population level. We calculated the time to loss of protective immunity for an individual as the time at which the titer reaches the threshold for protection using an exponential model for the waning of immunity.

Our approach is illustrated in Fig 1. First, we rescaled the antibody titer so that the threshold of protection was equal to 1, and we set the mean time for the time series for each individual to *t* = 0 (panel A). We then used a mixed-effects modeling framework to characterize the heterogeneity in antibody responses. This framework allowed us to determine the extent to which heterogeneity in responses depends on the vaccine or virus that induced the response, the individual (i.e., whether there were "strong responders" who tend to make high responses to all vaccines or viruses or who lose immunity more slowly than others in the population), and the interaction between these two effects. It also allowed us to quantify the heterogeneity in magnitude and decay rate of responses to each vaccine or virus and whether there was a relationship between the magnitude and decay rate (panel B). We then used a simple exponential decay model to determine the longevity of immunity in different individuals to different vaccine or virus antigens and quantify the loss of immunity to each vaccine or virus in the population (panel C).

## Materials and methods

### Data

We used data from a study that determined the antibody responses to a number of vaccine and viral antigens in a group of 45 individuals over a period of between 5 and 26 years, with a mean range of about 15.2 years (see [12] for details). These samples were taken as part of a center-wide, comprehensive program to permit serologic testing of people working in close proximity to nonhuman primates. We focused on the antibody responses to measles, rubella, vaccinia, mumps, and VZV antigens that were elicited either by a live-attenuated vaccine or natural infection, as well as antibodies to tetanus and diphtheria antigens that were elicited by immunization with inactivated protein toxin (i.e., toxoid) vaccines.

Because we wanted to consider the decay of antibody in the absence of boosting, the antibody data were curated to remove spikes due to revaccination or infection as described in more detail previously [12]. This involved censoring to exclude the following: timepoints for 3 years after immunization, when there was a rapid change in antibody levels [12,16], seronegative or unvaccinated individuals, and individuals with fewer than 4 contiguous data points. We then kept the time series with the largest number of contiguous data points for the response of each individual to each vaccine or virus antigen.

As previously described, antibody titers were measured using ELISA and calibrated when possible in terms of international units (IUs). This allowed us to rescale the antibody concentration by dividing it by the level at which protection is lost [8,17–21]. In our plots and analysis, the magnitude of responses is shown as the log of the scaled titer. We did not have a level at which protection is lost for mumps and VZV, and for these, we scaled against the threshold of detection for the ELISA assay for that antigen. The level of antibody required for protection for different infections was taken from the literature and was assumed to be the same for all individuals—we did not consider variation in the protective threshold between different individuals due to lack of relevant data (see Discussion). Note that we did not consider the absolute magnitude of the antibody response (e.g., moles/L or mg/mL) because the ELISA assays used do not measure this quantity.

Estimating the variability in the magnitude of the responses of different individuals to a given vaccine required taking into account uncertainty in the time of vaccination or infection and the different ages covered by the time series for different individuals. Because we do not find a significant correlation of antibody titer with age (and gender), but do find a strong correlation with time (see analysis in [12]), we used time rather than age as the main factor governing antibody titer. Because we did not know the time of vaccination, we shifted the time axis so that time equal to 0 corresponded to the mean age of the time series for each individual, and we used the intercept as a summary measure of the average magnitude of the response of the individual. We emphasize that the magnitude is not the peak magnitude just after vaccination or infection but rather the magnitude at the mean timepoint for that time series, which is expected to be many years or decades after vaccination or infection.

### Responses of different individuals to different vaccines

We used a mixed-effects model framework (Eq 1) to determine the contributions of different factors to the magnitude (scaled titer) and decay of the antibody response. The factors we considered are vaccine or virus antigen, the individual, and the interaction between these two.

We assumed that the loss of antibody for each individual follows an exponential decay so that the log of the antibody titer decays linearly with time [5,7,8,12]. We used a linear mixed-effects model (implemented using the lme4 package [22] in R [23]) described below.

$$\begin{array}{l} \log_{10}(Scaled\;Titer_{ij}(time))=\\ +a-b*time\leftarrow \mathrm{fixed}\;\mathrm{effects}\\ +u_{i}-v_{i}*time\leftarrow \mathrm{random}\;\mathrm{effects}\;\mathrm{for}\;\mathrm{vaccine}\;i\\ +u_{j}-v_{j}*time\leftarrow \mathrm{random}\;\mathrm{effects}\;\mathrm{for}\;\mathrm{individual}\;j\\ +u_{ij}-v_{ij}*time\leftarrow \mathrm{random}\;\mathrm{effects}\;\mathrm{for}\;\mathrm{vaccine}\;i\times \mathrm{individual}\;j\\ +e\leftarrow \mathrm{residual}\;\mathrm{for}\;\mathrm{the}\;\mathrm{model} \end{array}$$ (1)

*a* and *b* are the fixed, and *u*'s and *v*'s are the random effects for the magnitude and rate of decay. *u*_*i*_ and *v*_*i*_ tell us how much the response differs for different vaccines, *u*_*j*_ and *v*_*j*_ tell us how much an individual's response differs from the average response across all vaccines, and the terms *u*_*ij*_ and *v*_*ij*_ describe the interaction between vaccine and individual.

Statistical support for each random-effect parameter was determined by the extent to which its inclusion improved the Akaike Information Criterion (AIC) of the model [24]. Specifically, we determined ΔAIC = AIC of the model without that random effect minus AIC of the full model in Eq 1. The magnitude of ΔAIC indicates the measure of support for including the random effect, with 4<ΔAIC<10 indicating some support and ΔAIC>10 indicating strong support. We also checked the robustness of the results obtained using the mixed-effects model by repeating our analysis using a fixed-effects model and obtained similar results.

### Longevity of memory

We estimated the duration of immunity of an individual to each vaccine in the absence of boosting by calculating the time (relative to the mean of the time series for each individual) for immunity to fall to the threshold (taken from the literature) at which immunity is lost. Because we do not have data on variation in the protective level of immunity in different individuals, this threshold is assumed to be the same for all individuals. Prior studies suggest that, following an initial rapid decay following infection or vaccination, the magnitude of the antibody as well as T-cell responses wane exponentially with time [5,7,8,12]. As described earlier, the data were censored to exclude timepoints 3 years after immunization or infection when there is a rapid change in antibody levels. This allowed us to use an exponential decay model for the waning of antibodies. Let *M*_*ij*_ equal the magnitude of the response relative to protective titer and *D*_*ij*_ the rate of decay of the response for individual *j* to vaccine *i*; then the time, *T*_*ij*_, to loss of immunity is given by

$$T_{ij}=\frac{\log_{10}(M_{ij})}{D_{ij}}=\frac{a+u_{i}+u_{j}+u_{ij}}{b+v_{i}+v_{j}+v_{ij}}$$ (2)

## Results

**Fig 2:**
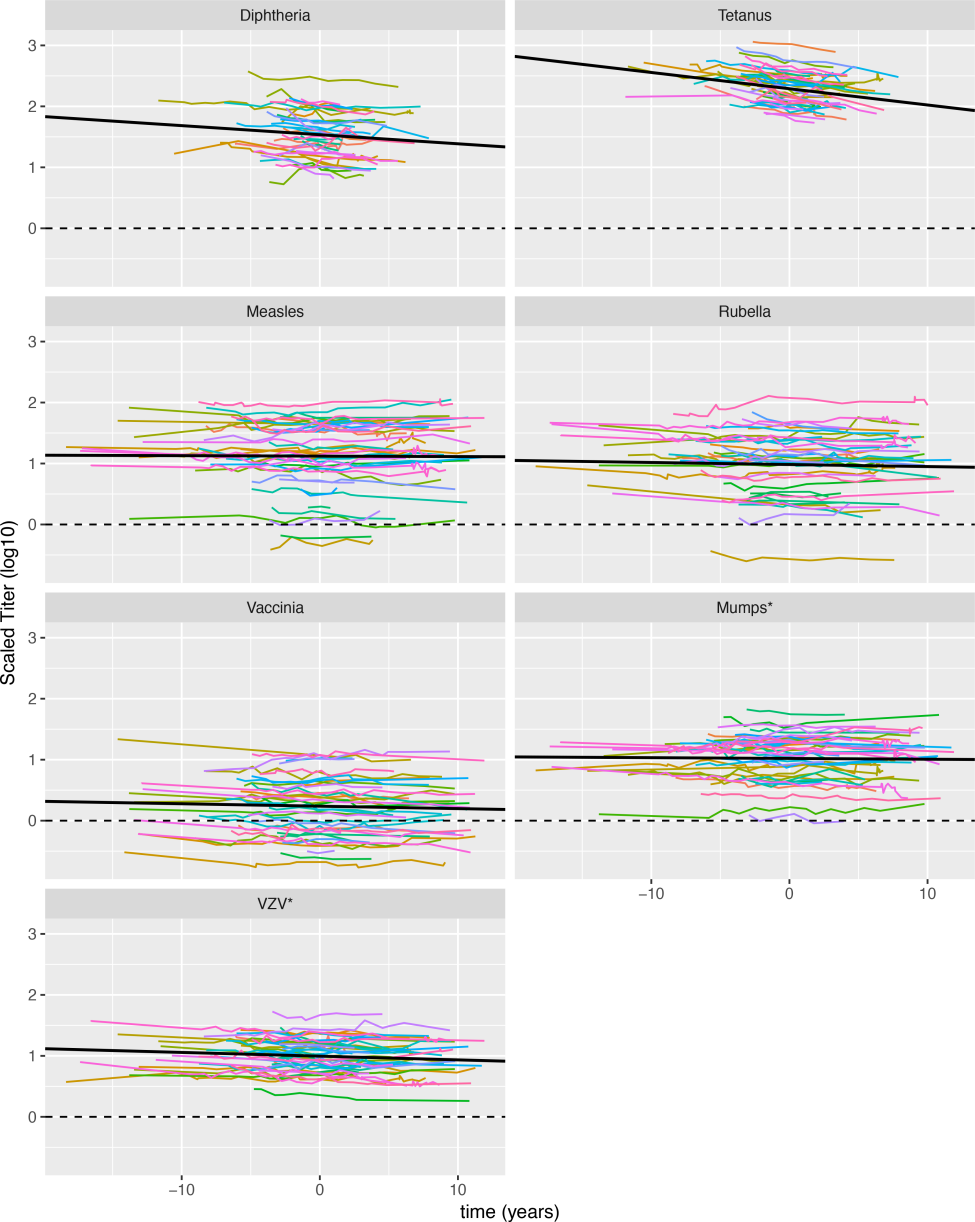
Plot of the rescaled data for the responses of individuals to different vaccine and virus antigens. Responses to each virus or vaccine antigen are shown in separate panels. The log of the scaled titer relative to the threshold for protection is plotted on the y-axis as a function of time with the mean of the time series for each individual shifted to time = 0. For mumps and VZV (marked with an asterisk), there is no standard for threshold for protection, and we use the threshold of detection. Underlying data for Fig 2 can be found on sheet Fig2_data in the S1 Data file. VZV, varicella zoster virus.

The curated data for the changes in antibody titer over time in different individuals to different vaccines are shown in S1 Fig, and the rescaled data are shown in Fig 2. The different panels show the responses to the different virus or vaccine antigens, and the different colors correspond to different individuals. The y-axis shows the logarithm of the antibody titer rescaled by dividing by the threshold for protection, and the x-axis shows time in years, with the mean of the time series for each individual assigned a value of time equal to 0.

### Contribution of different factors to antibody titers

We used a mixed-effects model framework described by Eq 1 to determine the contributions of different factors on the magnitude and rate of decay of antibody responses. The factors we considered were vaccine (or virus) antigen, the individual, and the interaction between these two. The results are shown in Table 1. The standard deviation (SD) indicates the amount of variation in the random effects for magnitude and decay rate for the relevant factor. This can be interpreted as follows: the random-effects model fits a line to each time series, and the variability in the magnitude and decay rate between these time series is partitioned between the 3 levels. Hence, for example, a large SD for vaccine magnitude (first line of Table 1) indicates large differences in the magnitude of responses from different vaccines.

**Table 1:** Table showing the contribution of different random effects on the variation in antibody responses.

Level or Factor	Random Effect	SD	Correlation *r*
Vaccine	Magnitude	0.760**	0.83 (ns)
	Decay rate	0.00961**	
Individual	Magnitude	0.200**	−0.86*
	Decay rate	0.00376*	
Vaccine × individual	Magnitude	0.385**	0.06 (ns)
	Decay rate	0.0103**	
Residual		0.0466	

The SD indicates the amount of variation in the relevant random effect, and *r* indicates the correlation between the variation in magnitude and decay rate for the level. We see that effects due to the vaccine, the individual, and their interaction account for most of the variation in antibody responses of different individuals to different vaccine and virus antigens. The asterisks indicate the extent to which inclusion of each factor improved the AIC: Δ*AIC*<4 is ns; 4<ΔAIC<10 (*) indicates some support; ΔAIC>10 (**) indicates strong support for including the factor [24]. The ΔAIC values are shown in S1 Table. The units for magnitude is log_10_ (scaled titer), and the decay rate is log_10_ (scaled titer) per year. Because we do not have values for the protective titer for mumps and VZV, we excluded these from the terms in the first line (corresponding to the random effect for vaccine magnitude). The results were obtained using the mixed-effects model described in Eq 1.

Abbreviations: AIC, Akaike Information Criterion; ns, no support; VZV, varicella zoster virus.

We see that all 3 factors (vaccine, individual, and vaccine × individual) contribute to the magnitude and decay rate of responses. The small residual indicates that, together, these factors account for most of the variation in the responses that are observed.

We compared the relative contributions of variation in the magnitude and variation in the decay rate to the variation observed. The SD of the decay rate in Table 1 corresponds to the variation arising per year. The ratio of the SD of the magnitude (units log_10_ [scaled titer]) to the SD of the decay rate (units log_10_ [scaled titer] per year) gives approximate time (in years) at which variation in the decay rate and magnitude of responses contribute comparably to the overall variation in the data (log_10_ [scaled titer]). This ratio is approximately 65, 52, and 38 for vaccine, individual, and vaccine × individual level effects, respectively. This indicates that, at times much less than 50 years, differences in magnitude contribute more than differences in decay rate of responses to variation in antibody titers. At times much greater than 50 years, differences in magnitude contribute less than differences in decay rate of responses to variation in antibody titers.

The largest effect is due to the vaccine level and indicates large differences in both the magnitude and the rate of decay of responses to different vaccine or virus antigens. Notably, tetanus has the highest magnitude, on average 195 times the threshold for protection, while responses to vaccinia are on average only 1.7 times the threshold for protection. Tetanus also has the fastest decay rate at 6.2% per year, while responses to measles exhibit virtually no decay (decay of 0.2% per year). The correlation between magnitude and decay rate arises because responses to toxoid vaccines (tetanus and diphtheria) have higher magnitude (relative to the threshold for protection) and decay rates compared with responses to live or attenuated viruses, but this correlation only improves the AIC of the model marginally (ΔAIC = 2.4) because of the small number of vaccine or virus antigens being considered.

The individual level also contributed significantly to the antibody titers in the mixed-effect model, though the effect size is modest. This showed that some individuals made higher responses on average to all vaccine or virus antigens than others, and the decay rate of antibody titers occurred slower in some than others (Table 1). With regard to the magnitude of responses, we estimate that an individual at the top 10th percentile would make about 3.2-fold higher response than an individual at the bottom 10th percentile. With regard to decay rates, we estimate that the response of an individual at the top 10th percentile would decay about 2.2% per year faster than an individual at the bottom 10th percentile. Furthermore, the negative correlation between the magnitude of the response of an individual and its rate of decay indicates that individuals who make higher responses on average have slower rates of loss of antibodies. This suggests that some individuals on average have larger, slower decaying antibody responses than others. The effect of variation in responses between individuals is also shown visually in S2 Fig, where we plot the relative magnitude and relative decay rates of the responses of different individuals.

Finally, the interaction term (vaccine × individual) has a large effect. It indicates the amount of variation in the magnitude and rate of decay that is not attributed to the vaccine and individual-level effects. We note the small SD associated with the residual, which suggests that the model fits the data well.

### Variability in responses of individuals to each vaccine

**Fig 3:**
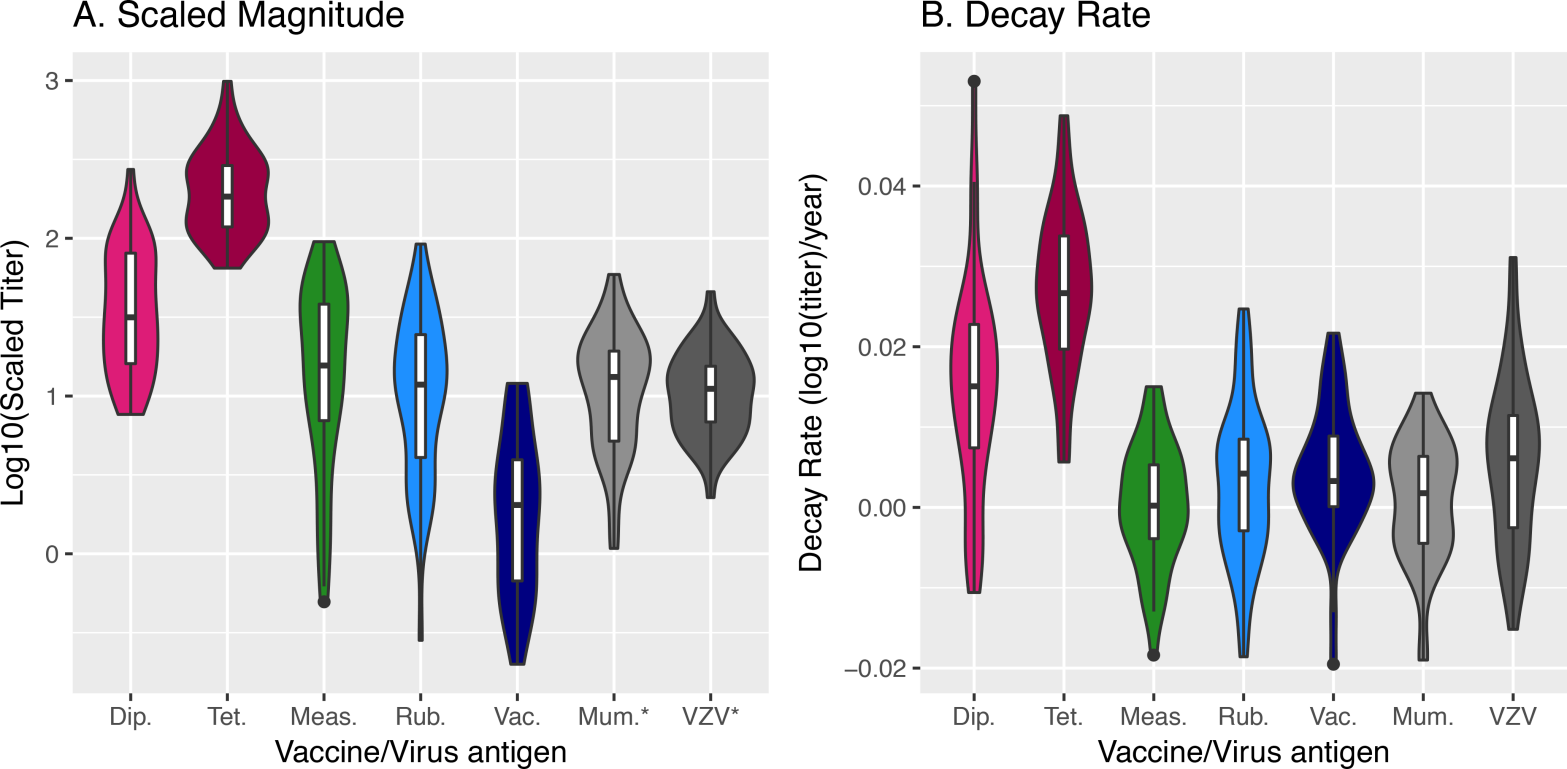
**We use a violin plot to describe the variation in magnitude (panel A) and decay rate (panel B) of the antibody responses to different vaccine or virus antigens.** In panel A, we plot the logarithm of the scaled titer of the antibody response. We note that antibodies to diphtheria and tetanus are induced by immunization with protein or toxoid antigens, and the responses to the other vaccines or viruses are induced by replicating viruses. The asterisk for mumps and VZV indicates that the protective titer has not been estimated for these vaccines, so only the variation and not the absolute magnitude of responses to these vaccines can be compared to other vaccines. Underlying data for Fig 3 can be found on sheet Figs 3 and 5, S3_data in the S1 Data file. Dip., diptheria; Meas., measles; Mum., mumps; Rub., rubella; Tet., tetanus; Vac., vaccinia; VZV, varicella zoster virus.

Fig 3 (and S2 Table) shows the average magnitude and decay rate of responses to different vaccine or virus antigens, as well as the extent of variation in these quantities. As has been previously noted [12], we found that antibody responses to diphtheria and tetanus (monovalent protein or toxoid antigen vaccines) decay faster than responses elicited to measles, rubella, and vaccinia (multivalent replicating virus vaccines). Our study suggests that this faster decay is compensated for by the responses to diphtheria and tetanus having a higher magnitude (measured relative to the threshold for protection) compared with responses to measles, rubella, and vaccinia.

In addition to different vaccines or viruses eliciting responses of different magnitudes, there is considerable variation in the response of different individuals to a given vaccine or virus antigen (Fig 3). We looked for correlations between the magnitude of the response an individual makes and the decay rate of the response. We found that for a given vaccine, individuals who made large responses, for the most part, tended to have slower decay rates, though for any given vaccine this trend did not reach statistical significance (at *p* = 0.05 level) (S3 Fig and S2 Table). The large negative correlation between the magnitude and decay rate associated with the individual-level effect across all vaccines seen in Table 1 arises due to the following: correlations between the magnitude of the response to one vaccine or virus antigen and the decay rate of responses to other vaccine or virus antigens, as well as the aggregation of weak correlations across multiple vaccine and virus antigens.

### Population-level immunity

We calculate the time to loss of protective immunity, defined as the time at which the antibody titer reaches the level defined to be protective for that vaccine or virus antigen. We assume that the antibody titer in an individual continues to decline exponentially. This allows us to estimate the time for the antibody titer in each individual to reach the level defined as the threshold for protection for the given virus or vaccine using Eq 2, namely, $T=\frac{\log_{10}(M)}{D}$, where *M* is the scaled titer at time equal to 0 and *D* is the decay rate for antibody concentration. We call the time until the antibody level reaches the defined threshold for protection for that pathogen the duration of immunity. Due to lack of data, we do not account for variation in the threshold of protection to a given pathogen in different individuals.

**Fig 4:**
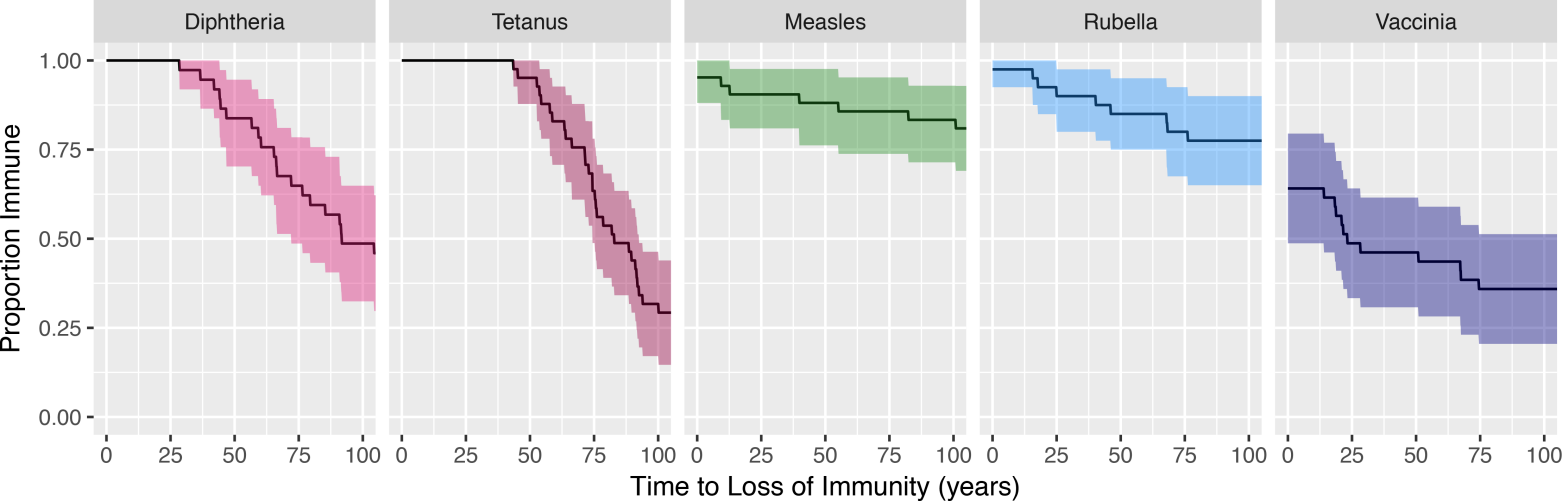
Loss of protective immunity. For each individual, we calculate the time to loss of protective immunity defined as the time before the antibody titer reaches the level defined to be protective for that vaccine or virus antigen (see Eq 2). We then plot the proportion of individuals who are immune as a function of time. The shaded areas represent the pointwise 95% CI obtained by bootstrap (1,000 replicates). Underlying data for Fig 4 can be found on sheet Fig4_data in the S1 Data file.

In Fig 4, we plot the proportion of the cohort immune to each vaccine or virus antigen as a function of time. As described in Materials and methods, we set time to 0 at the mean of each individual's time series in order to make comparisons between people of different ages. Year 0 corresponds on average to about 45 years in age. We found that virtually all individuals in this small cohort have protective levels of antibodies to tetanus, diphtheria, measles, and rubella at year 0, but only about two-thirds of individuals have protective levels of antibodies to vaccinia at this time.

We see different patterns for the loss of protective immunity elicited by protein or toxoid antigens (diphtheria and tetanus) versus live-attenuated vaccines or viruses (measles, rubella, and vaccinia). For both tetanus and diphtheria, it takes over 40 years for protective immunity to begin to be lost in our cohort. Thereafter, there is a relatively rapid loss of protective immunity, with about 7% and 12% of the population losing immunity to diphtheria and tetanus per decade. In contrast, immunity to measles and rubella begins to be lost sooner, but the decay rate of antibodies to these antigens is slower, with only 1% to 2% of the population losing protective immunity per decade. For vaccinia, just over 60% of the population have protective levels of antibodies at the outset (year 0), and this has dropped to 40% after 100 years—which corresponds to about 3% of the population losing protective immunity per decade.

## Discussion

We analyzed the data from a unique longitudinal study that quantified the level of antibodies to a panel of vaccine and virus antigens over a period of several decades in 45 individuals [12]. The original paper describing this dataset quantified the average rate of decay of antibodies to the different vaccine or virus antigens. In this paper, we extended this analysis in the following ways. First, we explicitly describe the heterogeneity in both magnitude (scaled to the threshold of protection) and decay rates of responses in different individuals to different vaccine and virus antigens (see Fig 3). We used a mixed-effects model to determine the contributions of different factors—vaccine, individual, and vaccine × individual—on antibody responses to different virus and vaccine antigens. This directly allows us to compare the extent to which the antibody response depends on the particular vaccine, the particular individual, and the interaction between these two. Second, we used a simple model to estimate the rates of loss of protective titers of antibodies to different vaccine and virus antigens at the population level.

**Fig 5:**
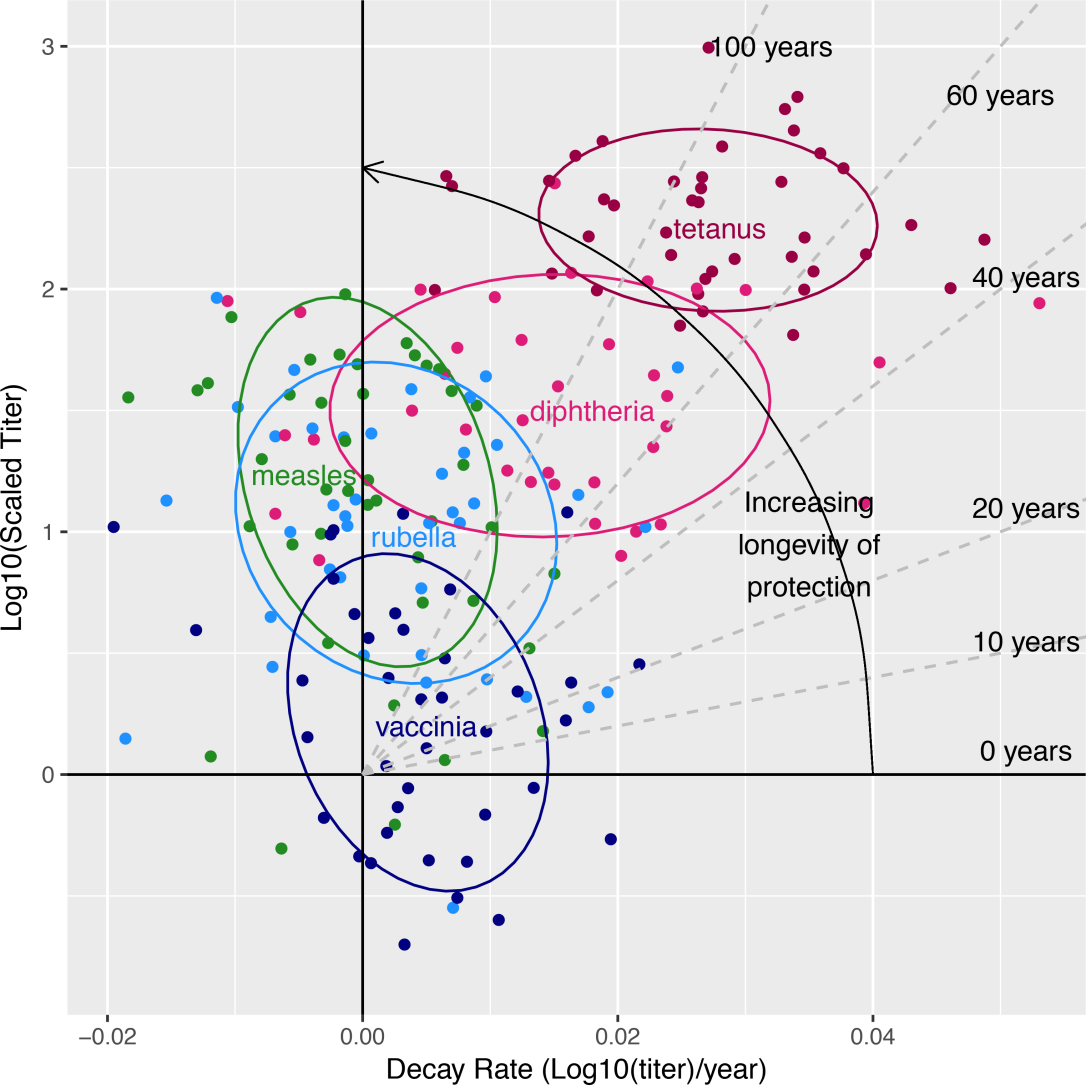
Summary plot that integrates the magnitude (i.e., log_10_ [scaled titer]), rate of decay (log_10_ [scaled titer] per year), and duration of protective immunity (years). The response to different vaccines or viruses is shown in different colors. For each vaccine or virus antigen, each point shows an individual's scaled titer and decay rate. The dashed lines demarcate regions with different durations of protective immunity. The ellipses represent the region encompassing 1 SD of the bivariate normal distribution for individuals to each vaccine. For example, we see that individual responses to tetanus have a higher magnitude and faster decay rates. The ellipse for tetanus indicates that the duration of protective immunity is between about 60 and 140 years for tetanus. In contrast, responses to measles have lower scaled magnitudes, but their decay rate is close to 0. Underlying data for Fig 5 can be found on sheet Figs 3 and 5, S3_data in the S1 Data file.

Our results are summarized in Fig 5, where the y-axis represents the magnitude of the response and the x-axis the rate of decay of the antibody response. The responses of different individuals to a given vaccine is represented by a cloud of points of a given color. The plot allows visualization of the variation in both the magnitude and the decay rate of responses of different individuals to a given vaccine, and it indicates how this variation translates into different times to loss of immunity shown by the dashed lines. The variation due to the factor "vaccine" can be seen by the separation between the clouds of points of different colors—for example, there is virtually no overlap between the responses to tetanus and measles. The extent of variation in the responses of different individuals to a given vaccine or virus antigen is indicated by the size of the cloud of points of each color. The extent of variation we observe is similar to that observed in other studies. For example, studies of antibody and T-cell responses following smallpox and yellow fever vaccination show similar variation [8,25].

We now describe the insights obtained for vaccination and boosting. Fig 5 also allows us to visualize the effects of increasing the magnitude (moving points up on the figure) or decreasing the decay rate (moving points to the left) on the time to loss of protective immunity to these infections. Given the ranges of variation in magnitude and decay rate seen in Fig 5, the duration of protective immunity to live-attenuated vaccines (measles, rubella, and vaccinia) could be best improved by focusing on increasing the magnitude of the responses to these vaccines. We see, for example, that increasing the magnitude of the response to vaccinia by 10-fold would be much more effective to provide long-term protection than decreasing the rate of decay. In contrast, the duration of protective immunity to tetanus and diphtheria might be best improved by lowering the rate of waning of immunity to these antigens rather than increasing the magnitude of these responses.

We observed that the fast rate of decay in responses to protein antigen vaccines (tetanus and diphtheria) was counteracted by the high magnitude of these responses relative to the levels needed for protection. This resulted in a similar fraction of the population having lost protective levels of antibodies after 50 years to tetanus, measles, diphtheria, and rubella (see Fig 4). This is a much longer timescale than the current boosting schedule for tetanus and supports the results of a much larger cross-sectional study of tetanus antibody titers [11].

The current study is an early step in the quantification of the longevity of memory to different vaccine and virus antigens in the human population. It has a number of limitations, and identifying these can aid in the design of future studies.

The study is restricted to the antibody response. This is because it was possible to get longitudinal measurements of the magnitude of antibody responses from sera that had been stored for over several decades. Obtaining similar data for the dynamics of T-cell responses is possible but more complicated because it requires isolation and cryopreservation of peripheral blood mononuclear cells (PBMCs) and long-term storage in liquid nitrogen.

Antibody levels to different vaccine and virus antigens were measured by their ELISA titer and normalized to the threshold of protection. The thresholds for protection were taken from the literature, and there are different standards for the protection to different infections [26,27]. We calculated the time for antibody titers to fall to the defined threshold for protection for the given vaccine or virus antigen. Due to a lack of data, we were not able to determine the consequences of variation in the threshold for protection between individuals. Different levels of immunity are required for different types of protection [27–30]. For example, higher levels of antibodies might be required to prevent infection, whereas lower levels of antibodies may not prevent infection per se but may still ameliorate disease or protect against lethal infection. Smallpox vaccination with vaccinia virus results in antibody titers that are predicted to provide full protective immunity against virulent poxvirus infection in about 40% of subjects at 100 years (Fig 4), and the remaining 60% of the population are likely to have partial protective immunity. This point was illustrated during the American monkeypox outbreak in 2003. Of 8 previously vaccinated individuals who were diagnosed with monkeypox, three-eighths had no clinical symptoms (monkeypox infection identified only through immunological assays), and the other five-eighths of vaccinated subjects had milder disease than that associated with unvaccinated monkeypox cases [31].

We did not have dates of prior vaccinations or infections, and some would occur prior to the period when the individuals were sampled. Because we were interested in antibody titers over the long term, we curated the data to eliminate a 3-year interval following a spike in antibody levels (see Materials and methods). Consequently, our results describe the duration of immunity in this population of adults rather than as a function of time since immunization. The lack of data on prior vaccinations also makes it difficult to consider how the number of prior exposures affects the level of boosting or changes in the decay rate of immunity. We note that antibody spikes were relatively infrequent (<0.3 events per 100 person-years) for vaccinia, mumps, and rubella, likely due to less frequent immunization as well as sterilizing immunity preventing boosting of immunity following vaccination with live-attenuated vaccine strains or natural infection. Antibody spikes following revaccination against tetanus and diphtheria (with protein toxoid antigens) were more common (4.9 events per 100 person-years). Finally, our extrapolation for the duration of immunity assumed that the rate of decay of antibody titers is constant and does not either increase (potentially due to negative effects of aging [32, 33]) or decrease (potentially due to "selection" for longer-lived plasma cells [15]) over time. We note that a cross-sectional study of antitoxin antibody responses suggests that antibody decay rates to tetanus and diphtheria are not significantly different in individuals under and over 50 years of age [11].

Overcoming the preceding limitations would require more detailed sampling just prior to and following immunizations, as well as extending the studies for a longer duration that incorporates older individuals.

## Conclusion

We examined the heterogeneity in both the magnitude and decay rate of antibody responses to different virus and vaccine antigens and used simple models to quantify how this heterogeneity affected the duration of protective immunity to a panel of vaccines and viruses. We found that variation in magnitude and decay rates of responses contribute comparably to the differences in antibody titers, that some individuals tend to make higher responses and these individuals also tend to have slower decay rates, and that different patterns of duration of protective levels of antibodies were elicited by replicating viruses and proteins. As noted in our previous study, we found that levels of antibodies to protein toxoid immunization to tetanus and diphtheria are higher relative to the protective threshold and decay faster than those elicited by replicating viruses. Integrating the effects of these two factors suggested that, in the absence of boosting, more adults will, for the first 4 decades, tend to have protective levels of antibodies to tetanus and diphtheria in comparison with measles, rubella, and vaccinia, suggesting the need for reevaluation of their boosting schedules. Our results emphasize the need for collecting longitudinal data that can allow quantification of the magnitude and decay of antibody titers in a large cohort of individuals to different vaccine and virus antigens, and we illustrate how integrating such measurements with models will allow us to generate a nuanced quantitative picture for the loss of protective immunity and help optimize boosting strategies.

## Supporting information

S1 Fig Shows all curated data used in analysis. The dotted lines indicate the threshold of protection. The y-axis is the log_10_ (ELISA titer). The boxplot at the bottom right indicates the number of points per individual response to each vaccine or virus antigen. The asterisk for mumps and VZV indicates the absence of a defined threshold for protection. Underlying data for S1 Fig can be found on sheet FigS1_data in the S1 Data file. VZV, varicella zoster virus.
(TIF)

S2 Fig **Plots of the relative magnitude (panel A) and decay rates (panel B) of responses in different individuals.** The relative magnitude of the response of each individual to a given vaccine or virus antigen is determined by comparing it to the average magnitude of the response to that vaccine or virus antigen and similarly for decay rate. Specifically, we divide the magnitude of the response of an individual by the mean magnitude of the response to that vaccine or virus antigen and divide by the SD, and for the decay rate, we subtract the mean decay rate for responses to that vaccine or virus antigen from the decay rate for that individual and divide by the SD. An ANOVA (*p* < 0.0001) indicates that some individuals have significantly different magnitudes (panel A) and decay rates (panel B) from others. Note that the order of the individuals is different for the two plots and goes from lowest magnitude (panel A) and lowest decay rate (panel B) to highest. Underlying data for S2 Fig can be found on sheet FigS2_data in the S1 Data file.
(TIF)

S3 Fig Correlation between the magnitude and decay rate of responses to vaccines. We plot the magnitude versus decay rate of each individual's response variation for each vaccine or virus antigen. We show the linear regression line and 95% pointwise CI for that line. While there appears to be a trend for higher-responding individuals to have lower rate of loss of antibody titers than lower-responding individuals, this does not reach statistical significance for individual vaccines (see S2 Table). Underlying data for S3 Fig can be found on sheet Figs **3** and **5**,S3_data in the S1 Data file.
(TIF)

S1 Table Summary table with AIC values showing that random effects due to the vaccine, the individual, and their interaction account for most of the variation in responses of different individuals to different pathogens. The SD indicates the amount of variation in the relevant random effect, and *r* indicates the correlation between the variation in magnitude and decay rate for the level. The units for magnitude is log_10_ (scaled titer), and the decay rate is log_10_ (scaled titer) per year. The Δ*AIC* indicates the extent to which inclusion of each factor improved the AIC. Because we do not have values for the protective titer for mumps and VZV, we excluded these from the terms in the first line (corresponding to magnitude for random-effect vaccine). The results were obtained using the mixed-effects model described in Eq 1. AIC, Akaike Information Criterion.
(PDF)

S2 Table Summary table for the responses to different vaccine and virus antigens. We show the average magnitude and decay rates of responses to the different vaccines and the extent of variation in these quantities. Because we do not have values for the protective titer for mumps and VZV, the mean magnitude of these cannot be compared to the responses to the other vaccine or virus antigens and is indicated by an asterisk (*). We found little correlation (*r*) between the magnitude and decay rate in the responses to a given vaccine (none significant). All results are from the mixed-effects model described in Eq 1. VZV, varicella zoster virus.
(PDF)

S1 Data The data for the Figs 2, 3, 4, 5, S1, S2 and S3 are in the relevant sheets of S1 Data. (XLSX)

S1 R scripts The R scripts used for the analysis of the data. These R scripts explain how to import the data from S1_data.xlsx file supplied and generate all the figures and tables in the paper. The PDF document contains both the scripts and shows the output for all the figures and tables in the manuscript generated by these scripts.
(PDF)

## Abbreviations

AIC: Akaike Information Criterion
IU: international unit
PBMC: peripheral blood mononuclear cell
SD: standard deviation
VZV: varicella zoster virus
