# Supplementary figures and images for: Heterogeneity and longevity of antibody memory to viruses and vaccines
Source: PLoS Biol. 2018 Aug 10;16(8):e2006601. doi: 10.1371/journal.pbio.2006601 (PMC6105026; doi:10.1371/journal.pbio.2006601)

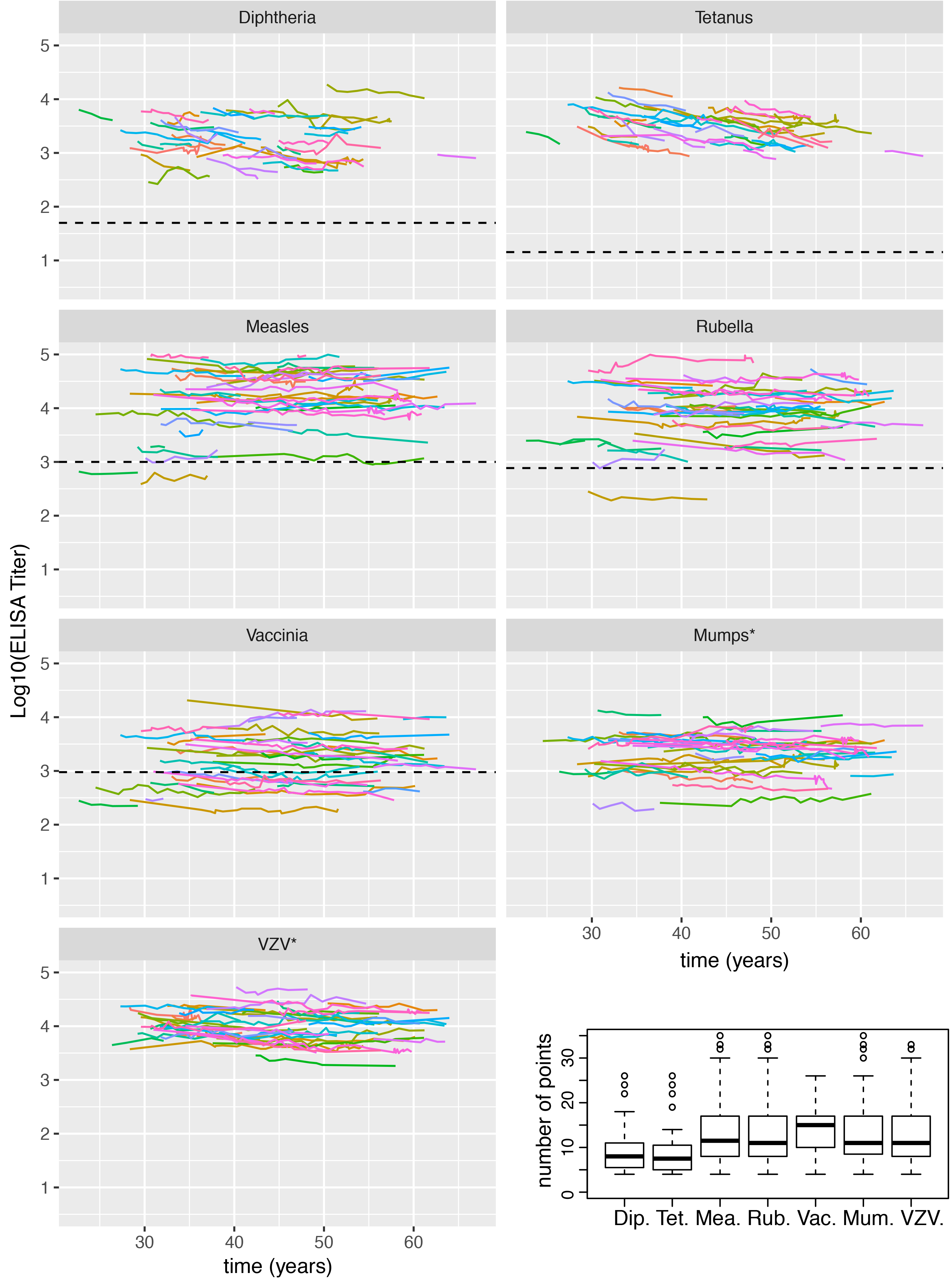

Supplement: S1 Fig — The dotted lines indicate the threshold of protection. The y-axis is the log10 (ELISA titer). The boxplot at the bottom right indicates the number of points per individual response to each vaccine or virus antigen. The asterisk for mumps and VZV indicates the absence of a defined threshold for protection. Underlying data for S1 Fig can be found on sheet FigS1_data in the S1 Data file. VZV, varicella zoster virus. (TIF) [file pbio.2006601.s001.tif]

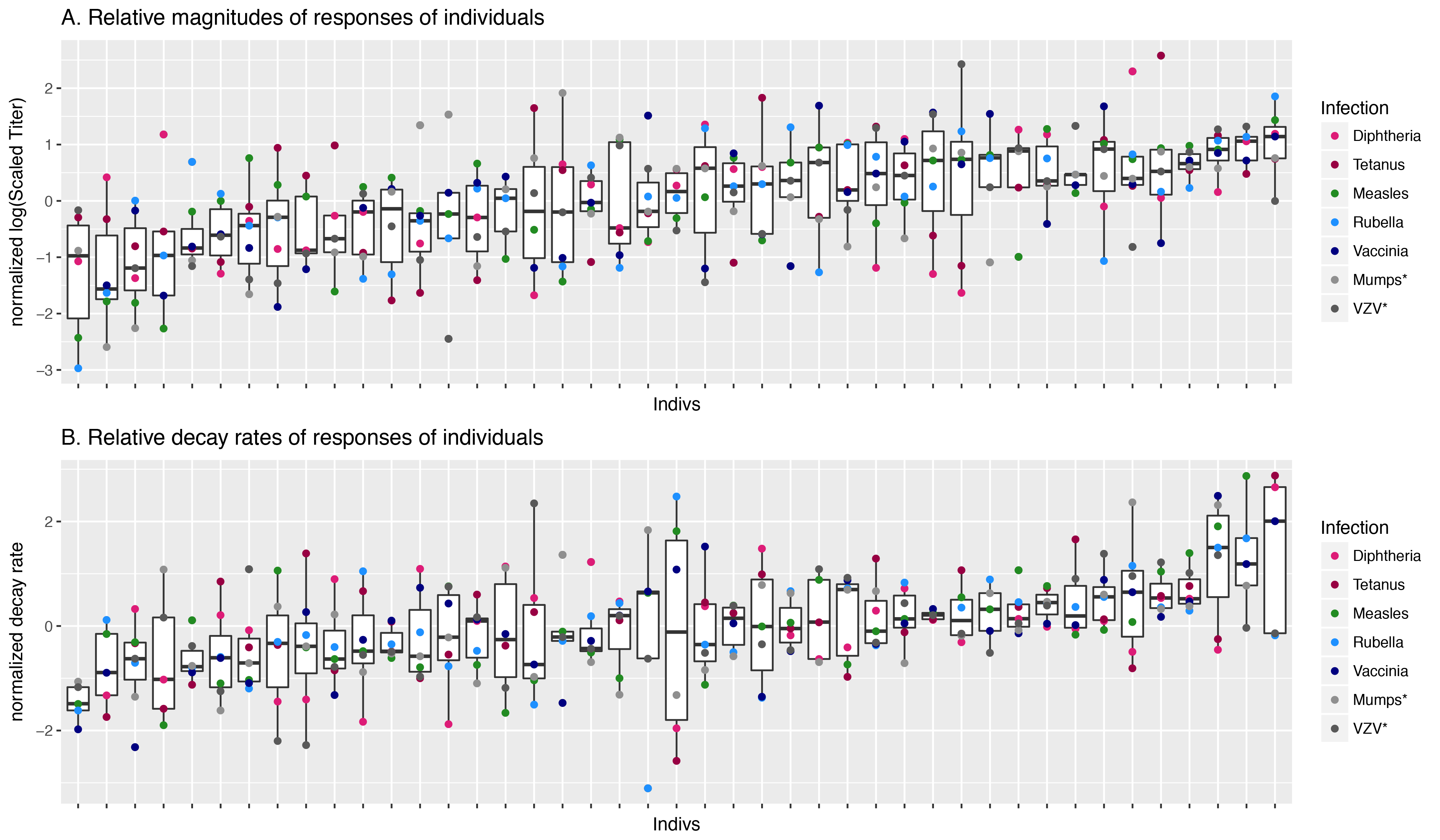

Supplement: S2 Fig — Plots of the relative magnitude (panel A) and decay rates (panel B) of responses in different individuals. The relative magnitude of the response of each individual to a given vaccine or virus antigen is determined by comparing it to the average magnitude of the response to that vaccine or virus antigen and similarly for decay rate. Specifically, we divide the magnitude of the response of an individual by the mean magnitude of the response to that vaccine or virus antigen and divide by the SD, and for the decay rate, we subtract the mean decay rate for responses to that vaccine or virus antigen from the decay rate for that individual and divide by the SD. An ANOVA (p < 0.0001) indicates that some individuals have significantly different magnitudes (panel A) and decay rates (panel B) from others. Note that the order of the individuals is different for the two plots and goes from lowest magnitude (panel A) and lowest decay rate (panel B) to highest. Underlying data for S2 Fig can be found on sheet FigS2_data in the S1 Data file. (TIF) [file pbio.2006601.s002.tif]

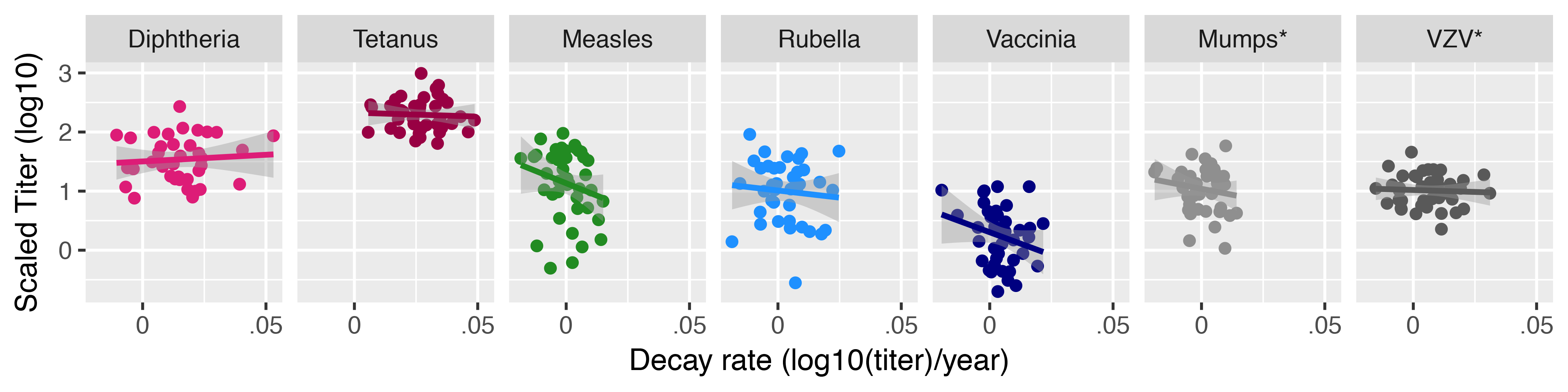

Supplement: S3 Fig — We plot the magnitude versus decay rate of each individual's response variation for each vaccine or virus antigen. We show the linear regression line and 95% pointwise CI for that line. While there appears to be a trend for higher-responding individuals to have lower rate of loss of antibody titers than lower-responding individuals, this does not reach statistical significance for individual vaccines (see S2 Table). Underlying data for S3 Fig can be found on sheet Figs 3 and 5,S3_data in the S1 Data file. (TIF) [file pbio.2006601.s003.tif]
