# Supplementary material for: Heterogeneity and longevity of antibody memory to viruses and vaccines
Source: PLoS Biol. 2018 Aug 10;16(8):e2006601. doi: 10.1371/journal.pbio.2006601 (PMC6105026; doi:10.1371/journal.pbio.2006601)
Supplement: S1 Table — The SD indicates the amount of variation in the relevant random effect, and r indicates the correlation between the variation in magnitude and decay rate for the level. The units for magnitude is log10 (scaled titer), and the decay rate is log10 (scaled titer) per year. The ΔAIC indicates the extent to which inclusion of each factor improved the AIC. Because we do not have values for the protective titer for mumps and VZV, we excluded these from the terms in the first line (corresponding to magnitude for random-effect vaccine). The results were obtained using the mixed-effects model described in Eq 1. AIC, Akaike Information Criterion. (PDF) [file pbio.2006601.s004.pdf]

Level or Factor	Random Effect	SD	Corr. r
vaccine	magnitude	0.760	0.83
		ΔAIC=220.7	ΔAIC=2.4
	decay rate	0.00961	
		ΔAIC=93.9	
individual	magnitude	0.200	-0.86
		ΔAIC= 24.5	ΔAIC= 5.8
	decay rate	0.00376	
		ΔAIC= 8.1	
vaccine × indiv.	magnitude	0.385	0.07
		ΔAIC= 11283	ΔAIC= -1.5
	decay rate	0.0103	
		ΔAIC=763	
residual		0.0466	
