# Supplementary material for: Heterogeneity and longevity of antibody memory to viruses and vaccines
Source: PLoS Biol. 2018 Aug 10;16(8):e2006601. doi: 10.1371/journal.pbio.2006601 (PMC6105026; doi:10.1371/journal.pbio.2006601)
Supplement: S2 Table — We show the average magnitude and decay rates of responses to the different vaccines and the extent of variation in these quantities. Because we do not have values for the protective titer for mumps and VZV, the mean magnitude of these cannot be compared to the responses to the other vaccine or virus antigens and is indicated by an asterisk (*). We found little correlation (r) between the magnitude and decay rate in the responses to a given vaccine (none significant). All results are from the mixed-effects model described in Eq 1. VZV, varicella zoster virus. (PDF) [file pbio.2006601.s005.pdf]

Vaccine or virus antigen	Scaled Magnitude Log_{10}(Scaled Titer)		Decay Rate $\log_{10}$(titer) per year		Correlation r (Magnitude vs. Decay Rate)
	mean	SD	mean	SD	
diphtheria	1.53	0.39	0.015	0.014	0.08
tetanus	2.29	0.27	0.027	0.010	-0.05
measles	1.12	0.59	0.00074	0.0076	-0.2
rubella	0.98	0.52	0.0034	0.098	-0.09
vaccinia	0.24	0.50	0.0040	0.0082	-0.3
mumps	1.02*	0.39	0.0013	0.0075	-0.2
VZV	1.0*	0.27	0.0061	0.010	-0.05
