# Supplementary material for: Heterogeneity and longevity of antibody memory to viruses and vaccines
Source: PLoS Biol. 2018 Aug 10;16(8):e2006601. doi: 10.1371/journal.pbio.2006601 (PMC6105026; doi:10.1371/journal.pbio.2006601)
Supplement: S1 R scripts — These R scripts explain how to import the data from S1_data.xlsx file supplied and generate all the figures and tables in the paper. The PDF document contains both the scripts and shows the output for all the figures and tables in the manuscript generated by these scripts. (PDF) [file pbio.2006601.s007.pdf]

R code for Heterogeneity and longevity of antibody memory to viruses and vaccines

1. R version 3.4.4
2. Rstudio version 1.1.442

```
rm(list=ls())
##Load Packages
#linear mixed effects models
    library(lme4)
#graphics
    library(ggplot2)
    library(gridExtra)
#apply finctions to dataframes
    library(plyr)
    library(dplyr)
```

Get data from the data files supplied by Nichole

To do so export the data from sheet FigS1_data of the S1_data.xlsx file as a .csv file called "curatedData.csv". Then set the correct working directory and import this data.

```
setwd("~/Dropbox/Antia-Slifka/results/") #set to appropriate working directory
all_vaccines <-  read.csv("curatedData.csv")
all_vaccines$indiv=as.factor(all_vaccines$indiv)
all_vaccines$infection <- factor(all_vaccines$infection,
        levels = c("Diphtheria", "Tetanus",
                  "Measles", "Rubella",
                  "Vaccinia", "Mumps*",
                  "VZV*"))

infection_name <- c("Diphtheria", "Tetanus",
                  "Measles", "Rubella",
                  "Vaccinia", "Mumps*",
                  "VZV*")

#Colors for graph

infection_colors <- c("#dd1c77", "#980043", "forestgreen",
                      "dodgerblue1", "navy", "gray56", "gray35")
```

Rescaling the data

1. We center each time series around its mean time point. To this end, we create the new variable "time" which marks the "time" in years since the mean time point of the time series for that individual infection combo.
2. We rescale the titer with respect to the protective titer.

```
#calculate time in respect to the mean time point
#for each individual vaccine combination
all_vaccines <- ddply(all_vaccines,.(indiv,infection),
```

```
                    function(x){x$time=x$age-mean(x$age); x})

#scale the measured titers by the protective titer and log the result (base 10)
all_vaccines$log_scaled_titer <-
    log10(all_vaccines$titer) - log10(all_vaccines$protective_titer)
```

Mixed effects model analysis

Run the model described below.

$$
\begin{aligned}
\log_{10}(\text{Scaled Titer}_{i,j}(\text{time})) = & \\
& + a - b * \text{time} && \leftarrow \text{fixed effect} \\
& + u_i - v_i * \text{time} && \leftarrow \text{random effect for vaccine } i \\
& + u_j - v_j * \text{time} && \leftarrow \text{random effect for individual } j \\
& + u_{ij} - v_{i,j} * \text{time} && \leftarrow \text{random effect for vaccine } i \times \text{individual } j \\
& + e && \leftarrow \text{residual for the model}
\end{aligned} \tag{1}
$$

NOTES:

1. Terminology:
    - vaccine === infection are used interchangably.
    - magnitude ==== intercept.
    - decay rate === -slope.
2. Run the full model with the data for all infections to estimate all effects EXCEPT for the intercept for infection and correlation between slope and intercept for infection.
    - Since we do not know the protective titers for mumps and vzv, run a 2nd model (data some_vaccines) without mumps and vzv to estimate intercept for infection and correlation between slope and intercept for infection.

```
# full model
infection.mem <- lmer(log_scaled_titer ~ time +
                   (time|infection) +
                   (time|indiv) +
                   (time|indiv:infection),
               data = all_vaccines)

#use all estimates for random effects except for the
#intercept for infection and correlation between slope and itercept for infection

# can output
#infection.mem
# or just table 1
VarCorr(infection.mem)
```

```
##  Groups          Name        Std.Dev.  Corr
##  indiv:infection (Intercept) 0.3852264
##                  time        0.0102817 -0.058
##  indiv           (Intercept) 0.1997049
##                  time        0.0037604 0.858
##  infection       (Intercept) 0.6282221
##                  time        0.0096112 -0.822
```

```
##  Residual                     0.0465796
```

```r
#remove mumps and VZV from data as we do not know the protective titers.
some_vaccines <- subset(all_vaccines,!infection%in%c("Mumps*","VZV*"))

someInfections.mem <- lmer(log_scaled_titer ~ time +
                     (time|infection) +
                     (time|indiv) +
                     (time|indiv:infection),
                data = some_vaccines)

#use  estimates for random effects for the
#intercept for infection and correlation between slope and intercept for infection
VarCorr(someInfections.mem)
```

```
##  Groups          Name        Std.Dev.  Corr
##  indiv:infection (Intercept) 0.4144359
##                  time        0.0111613 -0.063
##  indiv           (Intercept) 0.2227395
##                  time        0.0033841 1.000
##  infection       (Intercept) 0.7604477
##                  time        0.0110571 -0.834
##  Residual                    0.0475654
```

Calculate the AIC Values for Models without terms to see that we are not overfitting

```r
# AIC value for full model
AICfull=AIC(lmer(log_scaled_titer~ time +
          (1+time|infection) +
          (1+time|indiv) +
          (1+time|indiv:infection),
           data = all_vaccines))


# Delta AIC value for model lacking effects
-AICfull+AIC(lmer(log_scaled_titer~ time +
          #(1+time|infection) +       # <-- NO VACCINE LEVEL
          (1+time|indiv) +
          (1+time|indiv:infection),
           data = all_vaccines))
```

```
## [1] 338.315
```

```r
-AICfull+AIC(lmer(log_scaled_titer~ time +
          (0+time|infection) +        # <-- no vaccine magnitude effect
          (1+time|indiv) +
          (1+time|indiv:infection),
          data = all_vaccines))
```

```
## [1] 285.9227
```

```r
DeltaVaccDecay=-AICfull+AIC(lmer(log_scaled_titer~ time +
          (1|infection) +             # <-- no vaccine decay effect
          (1+time|indiv) +
          (1+time|indiv:infection),
```

```
         data = all_vaccines))

-AICfull+AIC(lmer(log_scaled_titer~ time +
          (1+time||infection) +     # <-- no corr. bet mag and decay for vaccine
          (1+time|indiv) +
          (1+time|indiv:infection),
         data = all_vaccines))
```

[1] 4.251211

```
-AICfull+AIC(lmer(log_scaled_titer~ time +
          (1+time|infection) +
          #(1+time|indiv) +       # <-- NO INDIVIDUAL LEVEL
          (1+time|indiv:infection),
           data = all_vaccines))
```

[1] 25.85304

```
DeltaIndivMag=-AICfull+
          AIC(lmer(log_scaled_titer~ time +
          (1+time|infection) +
          (0+time|indiv) +         # <-- no individual magnitude effect
          (1+time|indiv:infection),
         data = all_vaccines))

DeltaIndivDecay=-AICfull+
          AIC(lmer(log_scaled_titer~ time +
          (1+time|infection) +
          (1|indiv) +                 # <-- no individual slope effect
          (1+time|indiv:infection),
          data = all_vaccines))

DeltaIndivCorr=-AICfull+
          AIC(lmer(log_scaled_titer~ time +
          (1+time|infection) +
          (1+time||indiv) +           # <-- no corr bet mag and slope for individual effect
          (1+time|indiv:infection),
          data = all_vaccines))

-AICfull+AIC(lmer(log_scaled_titer~ time +
          (1+time|infection) +
          (1+time|indiv),
          #(1+time|indiv:infection), # <-- NO INDIVIDUAL x VACCINE LEVEL
           data = all_vaccines))
```

[1] 11281.87

```
DeltaVacXIndMag=-AICfull+
          AIC(lmer(log_scaled_titer~ time +
          (1+time|infection) +
           (1+time|indiv) +
          (0+time|indiv:infection),  # <-- no indiv. x vaccine magnitude effect
         data = all_vaccines))

DeltaVacXIndDecay=-AICfull+
          AIC(lmer(log_scaled_titer~ time +
```

```
          (1+time|infection) +
          (1+time|indiv) +
          (1|indiv:infection),       # <-- no indiv. x vaccine slope effect
         data = all_vaccines))

DeltaVacXIndCorr=-AICfull+
          AIC(lmer(log_scaled_titer~ time +
          (1+time|infection) +
          (1+time|indiv) +
          (1+time||indiv:infection),   # <-- no corr bet mag and slope for indiv. x vaccine
         data = all_vaccines))


# AIC value for model without mumps and VZV (used for vaccine level : magnitude and corr.)

AICsomeVaccines=AIC(lmer(log_scaled_titer~ time +
          (1+time|infection) +
          (1+time|indiv) +
          (1+time|indiv:infection),
           data = some_vaccines))

# AIC value for model lacking effects

DeltaVaccMag=-AICsomeVaccines+
          AIC(lmer(log_scaled_titer~ time +
          (0+time|infection) +        # <-- no vaccine magnitude effect
          (1+time|indiv) +
          (1+time|indiv:infection),
          data = some_vaccines))

DeltaVaccCorr=-AICsomeVaccines+
          AIC(lmer(log_scaled_titer~ time +
          (1+time||infection) +     # <-- no corr. bet mag and decay for vaccine
          (1+time|indiv) +
          (1+time|indiv:infection),
         data = some_vaccines))
```

Table S1: Delta AIC values

Factor	Mag	Slope	Corr
Vaccine	221	94	2.4
Indiv	25	8.1	5.8
Vacc X Indiv	11284	763	-1.4

Get estimates for the extent of variation due to each factor

Here we focus on the extent of variation in the magnitude of responses due to factor individual.

```
#NORMAL estimates
# get the SD for factor individual from infection.mem
indiv.mag.SD=as.data.frame(VarCorr(infection.mem))[4,5]
indiv.slope.SD=as.data.frame(VarCorr(infection.mem))[5,5]
```

```
deltaMag=10^(diff(qnorm(c(0.1,0.9),sd=indiv.mag.SD))) #90%/10% intercept RECOMMENDED
# 10^(diff(qnorm(c(0.05,0.95),sd=indiv.mag.SD))) #95%/5% intercept
deltaSlope=diff(qnorm(c(0.1,0.9),sd=indiv.slope.SD)) #90% - 10% slope RECOMMENDED
# diff(qnorm(c(0.05,0.95),sd=indiv.slope.SD)) #95% - 5% slope
deltaSlopePercent=100*log(10)*deltaSlope
```

1. SD for magnitude for level individual $= 0.2 \implies$ top 10 percentile would make a 3.2 fold larger response than the bottom 10 percentile.
2. SD for slope for level individual $= 0.0038 \implies$ the difference in slope for the top and bottom 10 percentile is about 0.0096. This corresponds to about 2.2 % percent difference in decay rate per year.

Make a dataframe with the magnitude and decay rate of each individual to each vaccine

We estimate the magnitude as $\log_{10}(M_{ij}) = a + u_i + u_j + u_{ij}$ and decay rate as $D_{ij} = b + v_i + v_j + v_{ij}$.

```
#get correlation for slope and intercept
#Put all coefficents for vacine indivaidual interaction in dataframes
estimates <- ranef(infection.mem)$`indiv:infection`

estimates <- cbind(estimates, matrix( data=unlist(strsplit(rownames(estimates), ":")),
        ncol=2, byrow=TRUE)) #parse id number and vaccine name

names(estimates)[3] <- paste("indiv")
names(estimates)[4] <- paste("infection")
names(estimates)[1] <- paste("inter_int")
names(estimates)[2] <- paste("inter_slope")

estimates$inf_int <- numeric(length(estimates$indiv))
estimates$inf_slope <- numeric(length(estimates$indiv))
estimates$id_int <- numeric(length(estimates$indiv))
estimates$id_slope <- numeric(length(estimates$indiv))

#random effect for vaccine
for(i in unique(all_vaccines$infection)){
    estimates[estimates$infection == i, "inf_int"] <-
        ranef(infection.mem)$infection[i, 1]
    estimates[estimates$infection == i, "inf_slope"] <-
        ranef(infection.mem)$infection[i, 2]
}
#random effect for individual
for(i in unique(all_vaccines$indiv)){
    estimates[estimates$indiv== i, "id_int"] <-
        ranef(infection.mem)$indiv[as.character(i), 1]
    estimates[estimates$indiv == i, "id_slope"] <-
        ranef(infection.mem)$indiv[as.character(i), 2]
}
#add all random effects together + fixed effect to get true estimate
estimates$mem_int <-
    estimates$inter_int + estimates$inf_int + estimates$id_int + fixef(infection.mem)[1]
estimates$mem_slope <-
    estimates$inter_slope + estimates$inf_slope +
    estimates$id_slope + fixef(infection.mem)[2]
```

```r
#select just the final coefficient
coefs=estimates[ ,c("indiv", "infection", "mem_int", "mem_slope")]

#put infectiones in
coefs$infection=factor(coefs$infection,
        levels=c('Diphtheria', 'Tetanus', 'Measles',
                 'Rubella', 'Vaccinia', 'Mumps*', 'VZV*'),
        labels =c('Diphtheria', 'Tetanus', 'Measles',
                  'Rubella', 'Vaccinia', 'Mumps*', 'VZV*'))

#make table with results for each vaccine from mem-
#the variation in each of the random effects and
#the correlation between slope and intercept (calulated from estimates - not done in mem)
infectionTable_mem<-coef(infection.mem)$infection
infectionTable_mem$infection<-rownames(infectionTable_mem)
infectionTable_mem$sdIntercept<- numeric(length(infectionTable_mem$infection))
infectionTable_mem$sdSlope <- numeric(length(infectionTable_mem$infection))
infectionTable_mem$corr <- numeric(length(infectionTable_mem$infection))
infectionTable_mem$pCorr <- numeric(length(infectionTable_mem$infection))
for(i in infectionTable_mem$infection){
    tmp = coefs[coefs$infection==i, ]
    infectionTable_mem[infectionTable_mem$infection==i, "sdIntercept"] <-sd(tmp$mem_int)
    infectionTable_mem[infectionTable_mem$infection==i, "sdSlope"] <-sd(tmp$mem_slope)
    infectionTable_mem[infectionTable_mem$infection==i, "corr"] <-
        cor(tmp$mem_int, tmp$mem_slope)
    infectionTable_mem[infectionTable_mem$infection==i, "pCorr"] <-
        summary(aov(tmp$mem_int~tmp$mem_slope))[[1]]$'Pr(>F)'[1]
}

infectionTable_mem$infection <- factor(infectionTable_mem$infection,
                    levels=c('Diphtheria', 'Tetanus', 'Measles',
                             'Rubella', 'Vaccinia', 'Mumps*', 'VZV*'))

print("Table S2")
```

```
## [1] "Table S2"
```

```r
infectionTable_mem
```

```
##            (Intercept)          time  infection sdIntercept     sdSlope
## Diphtheria   1.5350986 -0.0148943079 Diphtheria   0.3904824 0.013622439
## Tetanus      2.2891560 -0.0266700192    Tetanus   0.2727147 0.009937672
## Measles      1.1212471 -0.0007398653    Measles   0.5911759 0.007624475
## Rubella      0.9833554 -0.0033561367    Rubella   0.5204190 0.009811957
## Vaccinia     0.2371203 -0.0039834383   Vaccinia   0.4981840 0.008180360
## Mumps*       1.0202430 -0.0012640068     Mumps*   0.3852136 0.007529583
## VZV*         0.9951416 -0.0061274883       VZV*   0.2679385 0.010323721
##                   corr     pCorr
## Diphtheria -0.07852472 0.6441045
## Tetanus     0.04856842 0.7629950
## Measles     0.21681590 0.1678354
## Rubella     0.09137323 0.5749712
## Vaccinia    0.25324842 0.1198127
## Mumps*      0.15903109 0.3270055
```

```
## VZV*        0.05080627 0.7462781
```

Figure 2 and Supplementary Figure S1

Time course of dynamics of antibody in all individuals to each vaccine.

```
fig2= ggplot(data=all_vaccines, aes(x=time, y=log_scaled_titer, group=indiv, colour=indiv)) +
    geom_line() +
    facet_wrap( ~ infection, nrow=4) +
    geom_abline(data=infectionTable_mem, aes(slope=time,
                intercept=`(Intercept)`), size=.8)+
   geom_abline(aes(slope = 0, intercept=0), linetype=2)+
    theme(legend.position="none") +
    labs(x="time (years)", y="Scaled Titer (log10)")

figS1=ggplot(data=all_vaccines, aes(x=age, y=log10(titer), group=indiv, colour=indiv)) +
    geom_line() +
    facet_wrap( ~ infection, nrow=4) +
    geom_abline(data=infectionTable_mem, aes(slope=time,
                intercept=`(Intercept)`), size=.8)+
   geom_abline(aes(slope = 0, intercept=0), linetype=2)+
    theme(legend.position="none") +
    labs(x="time (years)", y="Scaled Titer (log10)")

# inset for figS1
# counts number of datapoints for each individual for each vaccine
numPts <- ddply(all_vaccines,.(indiv,infection),
                   function(x){x$numPts = length(x$age); x}) %>%
    select("indiv", "infection", "numPts") %>% unique()

figS1npts=ggplot(data = numPts, aes(x = infection, y =numPts)) +
        geom_boxplot()+
        scale_x_discrete(labels=c('Dip.', 'Tet.', 'Meas.',
                           'Rub.', 'Vac.', 'Mum.*', 'VZV*'))
```

Plot Figure 2 (and output to file).

```
fig2  # plot
```

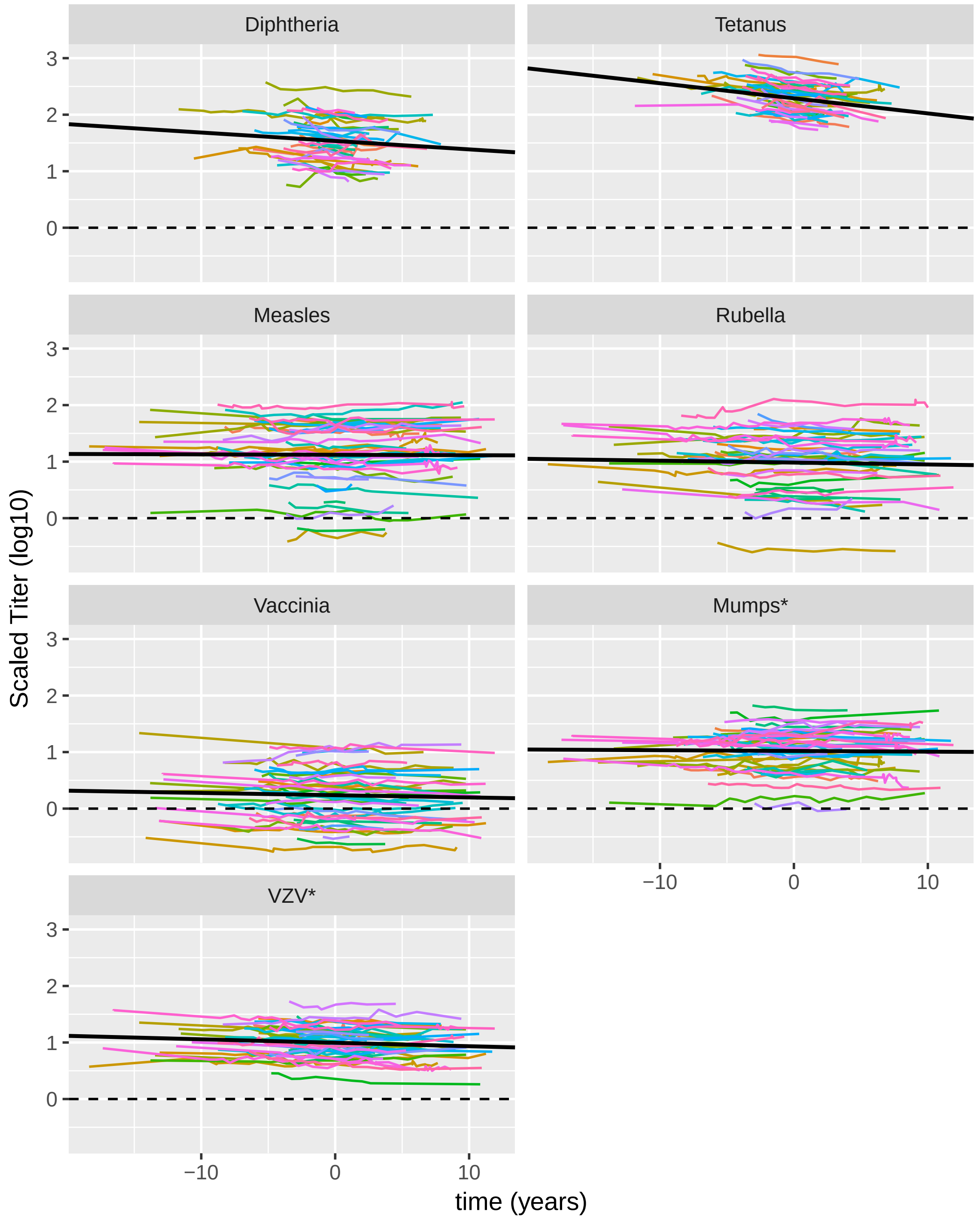


```
# export to file
pdf("Fig2.pdf", height = 10, width = 8)
fig2
dev.off()
```

```
## pdf
##   2
```

Plot Figure S1 (and output to file).

```
figS1
```

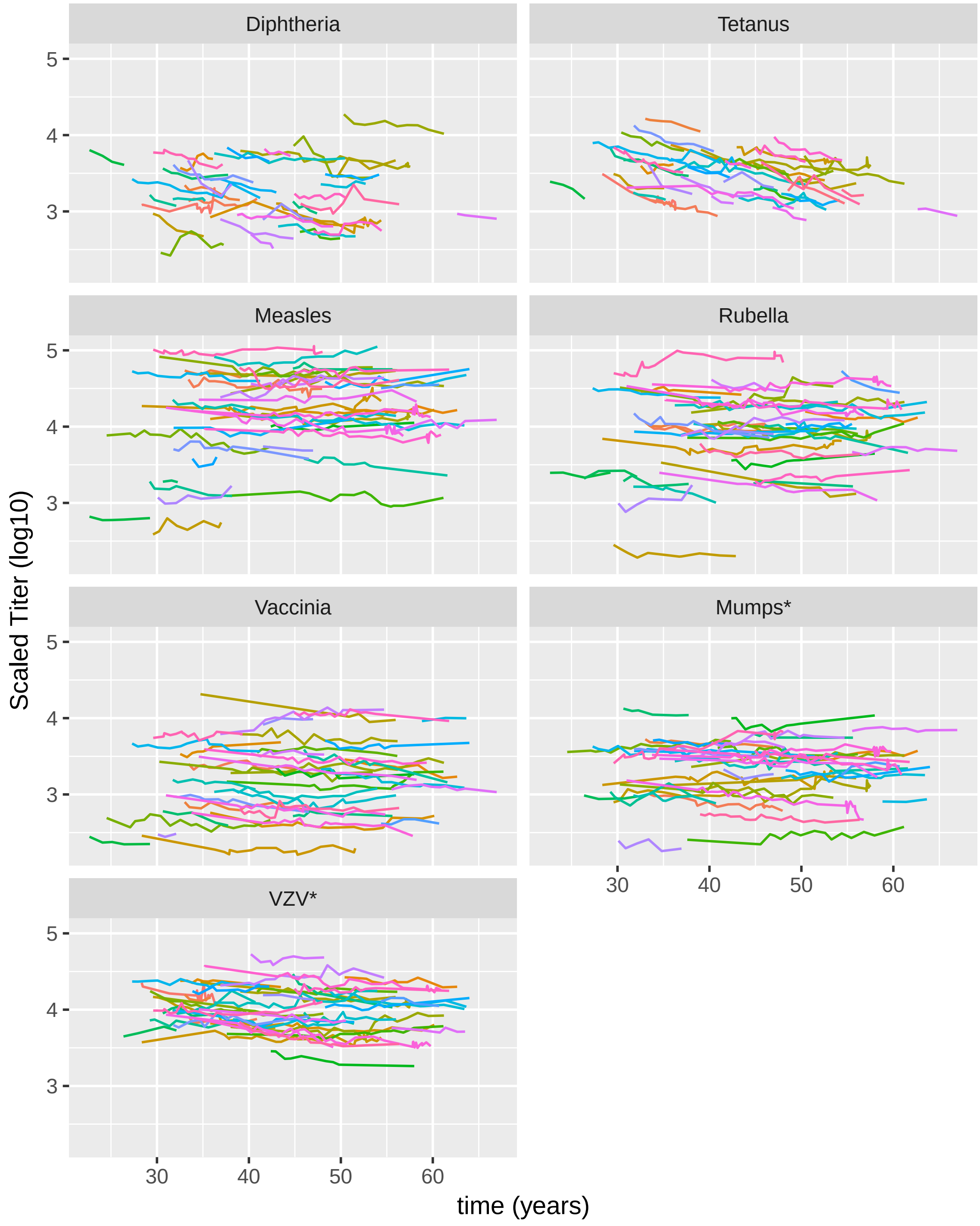


```
# export to file
pdf("S1_fig.pdf", height = 10, width = 8)
figS1
dev.off()
```

```
## pdf
##   2
```

Plot Figure S1 inset (and output to file).

```
figS1npts
```

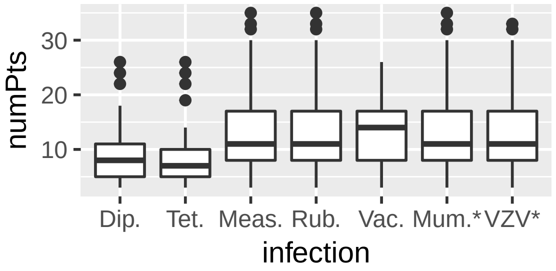


```
# export to file
pdf("S1_fig_npts", height=2,width=4)
figS1npts
dev.off()
```

```
## pdf
##   2
```

Figure 3 and Supp Figure 3

In Figure 3 plot the variation in Magnitude and Decay rate and in Supp Figure 3 the corr between mag and decay rate for each vaccine.

```
#plot intercepts from mems
fig3a <- ggplot(data=coefs, aes(x=infection, y=mem_int, na.rm=TRUE)) +
    geom_violin(aes(fill=infection)) + geom_boxplot(width=.1) +
    scale_fill_manual(values=infection_colors)+
    scale_x_discrete(labels=c('Dip.', 'Tet.', 'Meas.',
                            'Rub.', 'Vac.', 'Mum.*', 'VZV*')) +
    ggtitle("A. Scaled Magnitude") +
    xlab('Vaccine/Virus antigen') + ylab('Log10(Scaled Titer)') +
    guides(fill=FALSE) #+ theme_gray(base_size=14)


#plot slopes from mems
fig3b <- ggplot(data=coefs, aes(x=infection, y=-mem_slope, na.rm=TRUE )) +
   geom_violin(aes(fill=infection)) + geom_boxplot(width=.1) +
   scale_fill_manual(values=infection_colors) +
   ggtitle("B. Decay Rate")+
   scale_x_discrete(labels=c('Dip.', 'Tet.', 'Meas.',
                            'Rub.', 'Vac.', 'Mum.', 'VZV')) +
    xlab('Vaccine/Virus antigen') + ylab('Decay Rate (log10(titer)/year)') +
    guides(fill=FALSE)  # + theme_gray(base_size=16)

#plot correlation from mems
figS3 <- ggplot(data=coefs, aes(y=mem_int, x=-mem_slope, color=infection, na.rm=TRUE )) +
    scale_x_continuous(breaks = c( -.05, 0, .05, .1),
                    labels = c("-.05", "0", ".05", ".1")) +
    geom_point(size=1.5) +
    geom_smooth(method="lm", level=.95)+
    scale_color_manual(values=infection_colors)+
    facet_wrap( ~ infection, nrow=1) +
```

```
    ylab('Scaled Titer (log10)') + xlab('Decay rate (1/year)') +
    guides(fill=FALSE) + theme(legend.position="none") # + guidestheme_bw()
```

Plot Figure 3.

```
# To view figures
grid.arrange(fig3a,fig3b,nrow=1)
```

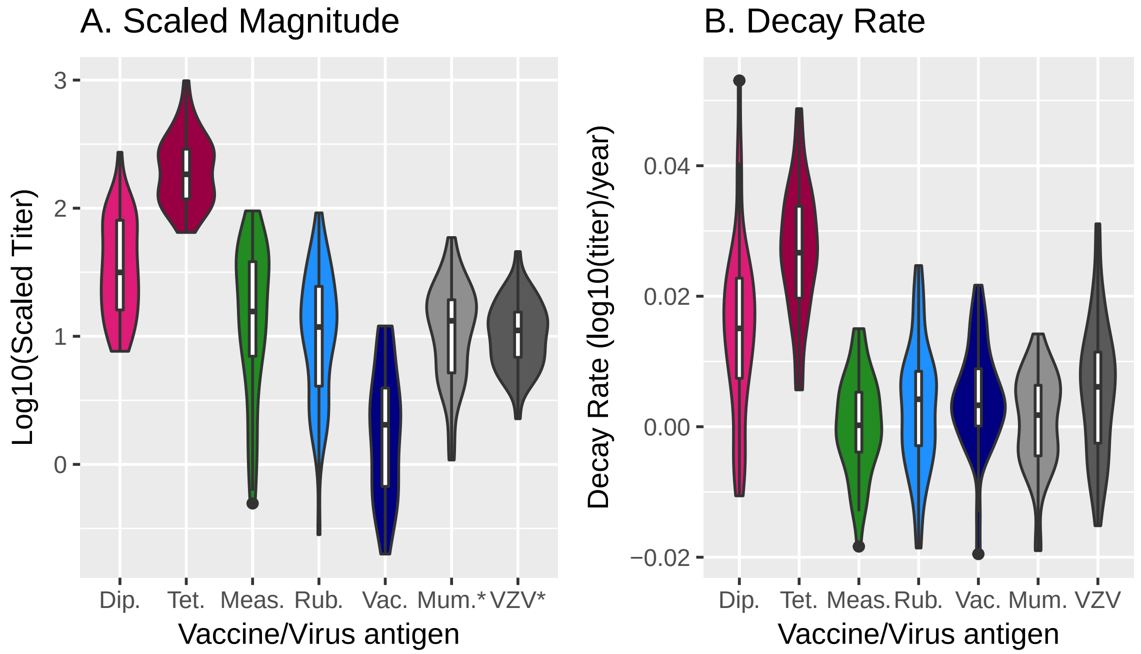


```
# export to pdf
pdf("Fig3.pdf", height = 4, width = 8)
  grid.arrange(fig3a,fig3b,nrow=1)
dev.off()
```

```
## pdf
##   2
```

Plot figure S3.

```
figS3
```

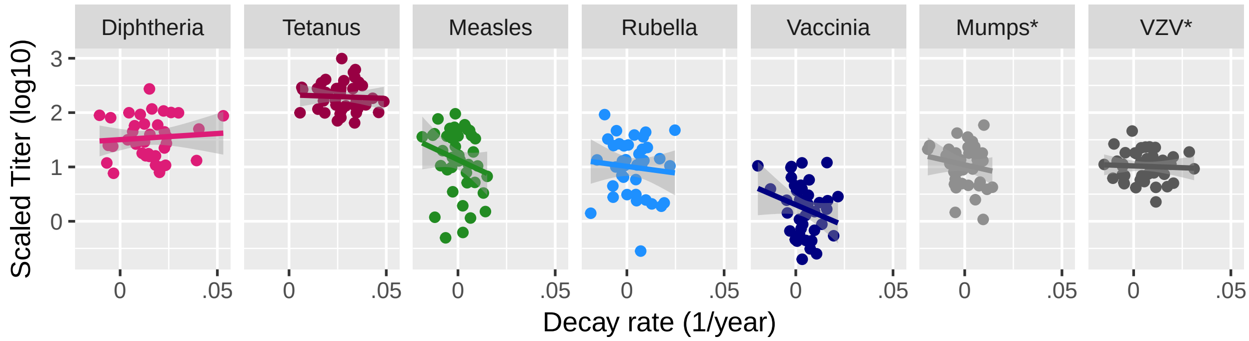


```
# export Supp Fig3
pdf("S3_fig.pdf", height = 2, width = 8)
 figS3
dev.off()
```

```
## pdf
##   2
```

Calculations for time to loss

Caclculate the time to loss of protective titers of antibodies for each individual to each vaccine.

```
coefs$mem_loss = numeric(length(coefs$indiv))
#find the expected time for loss of immunity for each indiv to each vaccine
for (i in 1:length(coefs$indiv)){
     #they have already lost immunity
    if(coefs$mem_int[i] <= 0) {coefs$mem_loss[i] = -1
    #slope is positive so they will never loss imunity
    } else if (coefs$mem_slope[i] >= 0) {coefs$mem_loss[i] = 10000
    #expected time of loss
    } else {coefs$mem_loss[i]= -coefs$mem_int[i]/coefs$mem_slope[i]}
}

ecdf_data_mem = data.frame(time=integer(0), Infection=character(0), prop=numeric(0))

#now calculate what proportion of the individiuls have lost protoection at some time t.
for (j in 1:7){
    temp = coefs[coefs$infection == infection_name[j], ]
    for (i in 0:1001) {
        prop = mean (temp$mem_loss <=i)
        t = data.frame(time = i, Infection= infection_name[j], prop)
        ecdf_data_mem = rbind(ecdf_data_mem, t)
    }
}

ecdf_data_mem1=ecdf_data_mem[ecdf_data_mem$Infection!="Mumps*"
                             &ecdf_data_mem$Infection!="VZV*", ]
```

Boostrap for error estimates

To get errors on time to loss plot run a bootstrap … change n to run the required number of bootstraps. (beware can take a LONG time (hours for thousands of replicates .. we use 1000 replicates in the paper))

```
# eval=FALSE prevents it running each time ...

boot_data<-NULL
for(j in 1:5){
    print(infection_name[j])
    infection <- coefs[coefs$infection==infection_name[j], c("indiv", "mem_loss")]
    times <- c(0, unique(infection$mem_loss) + 0.1, unique(infection$mem_loss) -0.1)
    n <-length(infection$indiv)
    for(i in 1:100){                # number of replicates for bootstrap here
        boot <- infection[sample(n, n, replace=T), ]
        for(t in times){
            prop = mean (boot$mem_loss <=t)
            temp_row <- data.frame(Infection=infection_name[j],
                                   Time = t, prop, bootNumber=i)
            boot_data<-rbind(boot_data, temp_row)
```

```
        }
    }
}
```

```
## [1] "Diphtheria"
## [1] "Tetanus"
## [1] "Measles"
## [1] "Rubella"
## [1] "Vaccinia"
```

Quantiles from the bootstrap.

```
quantiles <- NULL

for(j in 1:5){
    infection <- coefs[coefs$infection==infection_name[j], c("indiv", "mem_loss")]
    times <- c(0, unique(infection$mem_loss) + 0.1, unique(infection$mem_loss) -0.1)
    for(t in times){
        trueProp <- mean(infection$mem_loss<=t)
        tempData<-boot_data[boot_data$Infection==infection_name[j] &
                              round(boot_data$Time, 4)==round(t,4),  ]
        tempRow <- data.frame(Infection=infection_name[j], time = t, trueProp,
                             quant975 = quantile(tempData$prop, .975),
                             quant025 = quantile(tempData$prop, .025))
        quantiles <- rbind(quantiles, tempRow)
    }
}
```

Generate Figure 4: time to loss of protective titers for each vaccine

```
fig4=ggplot(data=quantiles[quantiles$time>=0, ], aes(x = time, y=1 - trueProp, na.rm=TRUE)) +
    geom_line()+
    geom_ribbon(aes(ymin=1-quant975, ymax=1-quant025, fill=Infection), alpha=0.4) +
    scale_fill_manual(values=infection_colors)+
    coord_cartesian(xlim=c(0, 100)) +
    facet_wrap(~Infection, nrow=1) +
    ylab('Proportion Immune') + xlab('Time to Loss of Immunity (years)') +
    theme_gray() +
    theme(legend.position="none")
```

Plot Figure 4.

```
fig4
```

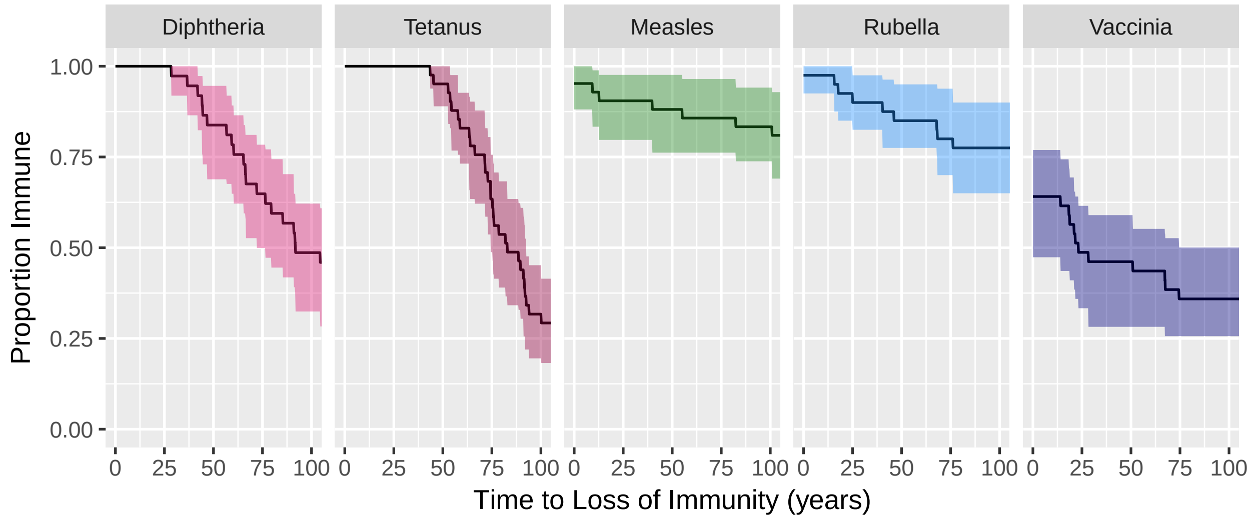


```
# export to pdf
pdf("Fig4.pdf", height = 3, width = 9)
dev.off()
```

```
## pdf
##   2
```

Summary Figure (Figure 5)

Summary graph with ellipses for each vaccine.

```
coefs_1=droplevels(coefs[coefs$infection!="Mumps*" & coefs$infection!="VZV*", ])
names(coefs_1)[2]="Infection"

unique(coefs_1$Infection)
```

```
## [1] Diphtheria Tetanus    Measles    Rubella    Vaccinia
## Levels: Diphtheria Tetanus Measles Rubella Vaccinia
```

```
fig5=ggplot(data=coefs_1, aes(x=-mem_slope, y=mem_int, na.rm=TRUE )) +
    geom_point(aes(colour= Infection)) +
    stat_ellipse(aes(colour= Infection),level=.68) +
#      stat_ellipse(aes(colour= Infection), level=.95) +
    scale_colour_manual(values=infection_colors)+
    geom_vline(xintercept=0) + geom_hline(yintercept=0)+
geom_segment(x=0,y=0,xend=.1,yend=1, color="grey",size=.5,lty=2)+
geom_segment(x=0,y=0,xend=.1,yend=2, color="grey",size=.5, lty=2)+
geom_segment(x=0,y=0,xend=.1,yend=4, color="grey",size=.5,lty=2)+
geom_segment(x=0,y=0,xend=.1,yend=6, color="grey",size=.5, lty=2)+
geom_segment(x=0,y=0,xend=.1,yend=10, color="grey",size=.5, lty=2)+
geom_curve(x=0.04,y=0,xend=0,yend=2.5,
           color="black",size=.3,curvature=.4,arrow=arrow(length=unit(0.3,"cm")))+
annotate("text",x=.028,y=2.25,label="tetanus", color=infection_colors[2],size=4)+
annotate("text",x=.018,y=1.5,label="diphtheria", color=infection_colors[1],size=4)+
annotate("text",x=-.0046,y=1.235,label="measles", color=infection_colors[3],size=4)+
annotate("text",x=.004,y=.98,label="rubella", color=infection_colors[4],size=4)+
annotate("text",x=.005,y=.2,label="vaccinia", color=infection_colors[5],size=4)+
annotate("text",x=.055,y=0.1,label="0 yrs", size=4)+
```

```
annotate("text",x=.055,y=.55,label="10 yrs", size=4)+
annotate("text",x=.055,y=1.1,label="20 yrs", size=4)+
annotate("text",x=.055,y=2.05,label="40 yrs", size=4)+
annotate("text",x=.05,y=2.8,label="60 yrs", size=4)+
annotate("text",x=.032,y=3,label="100 yrs",size=4)+
annotate("text",x=.035,y=0.95,label="Increasing\nlongevity of\nprotection",size=4)+
    ylim(-0.8, 3) +
    xlab('Decay Rate (Log10(titer)/year)') + ylab('Log10(Scaled Titer)')+
    theme(legend.position="none")
```

Plot figure 5.

```
fig5
```

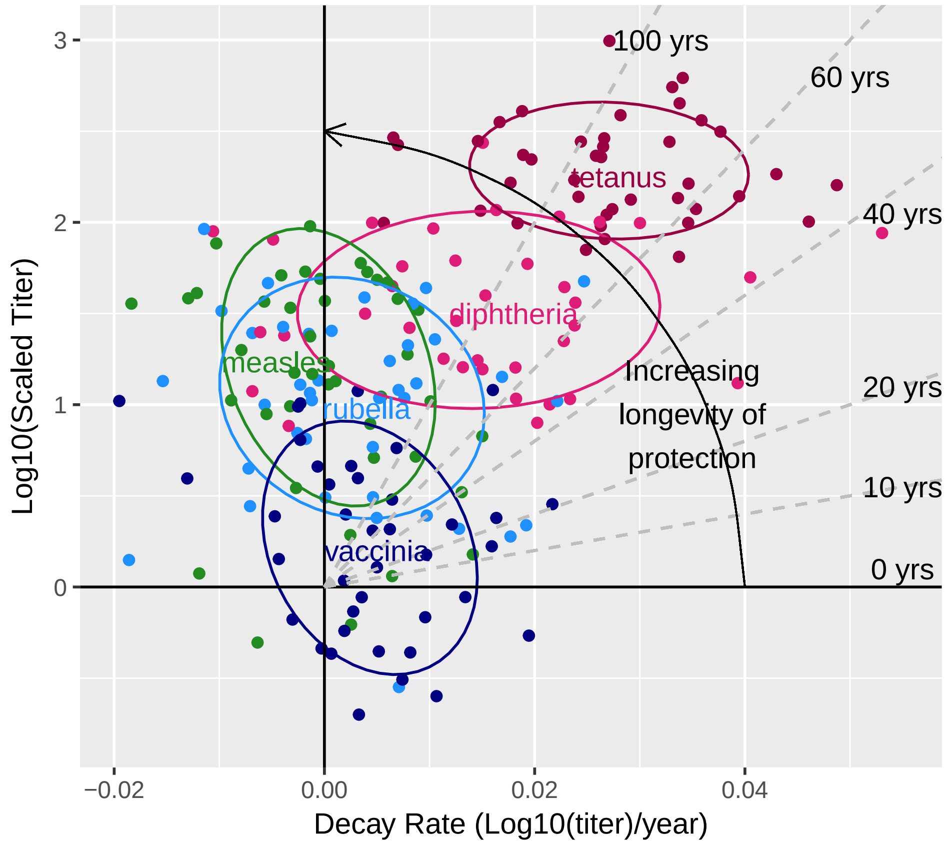


```
pdf("Fig5.pdf", height = 6, width = 6)
  fig5
dev.off()
```

```
## pdf
##   2
```

Fixed effects analysis

Make a dataframe with the magnitude and decay rate of each individual to each vaccine using individial linear regressions for each individual vaccine combo. Note: using this data frame you can make all the figures shown for the mixed effects results (except the 1st one) by substituting the appropriate variable names.

```
#make empty variables in coefs dataframe
coefs$fixed_int = numeric(length(coefs$indiv))
coefs$fixed_slope = numeric(length(coefs$indiv))

#Get a slope and intercept for each infection: indiv combination
#put in table with coeficients from mem for each infection
for (i in 1:7){
    temp_v =all_vaccines[all_vaccines$infection == infection_name[i], ]
    for (j in unique(temp_v$indiv)){
        temp_i = temp_v[temp_v$indiv == j, ]
        lm = lm (log_scaled_titer ~ time, data = temp_i)

        coefs[coefs$infection == infection_name[i] & coefs$indiv == j, "fixed_int"] <-
            coef(lm)[[1]]

        coefs[coefs$infection == infection_name[i] & coefs$indiv == j, "fixed_slope"] <-
            coef(lm)[[2]]
    }
}

#make table with results from all fixed effects
infectionTable_fix = data.frame(infection = character(0),
                                mag_mean= numeric(0), mag_sd = numeric(0),
                                slope_mean =numeric(0), slope_sd =numeric(0),
                                corr= numeric(0), p_corr= numeric(0))

for(i in 1:5){
    tmp = coefs[coefs$infection == infection_name[i], ]
    lm = lm(fixed_int ~ fixed_slope, data = tmp)

    newRow = data.frame(infection = infection_name[i], mag_mean=mean(tmp$fixed_int),
                        mag_sd=sd(tmp$fixed_int),
            decay_mean=mean(tmp$fixed_slope), decay_sd=sd(tmp$fixed_slope),
            corr=cor(tmp$fixed_int, tmp$fixed_slope),
            p_corr=summary(lm)$coefficients[2,4])

    infectionTable_fix = rbind(infectionTable_fix, newRow)
}
```

Fixed effects ANOVA

Keep individuals who make responses to x or more vaccines.

```
#calculate mean  of intercept and slope for each vaccine
coefs=ddply(coefs,.(infection), function(x)
        {x$mean_fixed_int=mean(x$fixed_int);
        x$mean_fixed_slope=mean(x$fixed_slope); x})

#calculatestandard deviation of intercept and slope for each vaccine
coefs=ddply(coefs,.(infection), function(x)
        {x$sd_fixed_int=sd(x$fixed_int);
        x$sd_fixed_slope=sd(x$fixed_slope); x})
```

```r
#calculate the number of vaccines each indivudual has data for
coefs = ddply(coefs,.(indiv), function(x)
        {x$numInfections=length(x$fixed_int); x})

#Choose min number of vaccines per person to include in model
x = 5

coefsX = coefs[coefs$numInfections >= x, ]

#run ANOVA on magnitude
aov_mag=aov((fixed_int-mean_fixed_int)/sd_fixed_int ~ indiv, data= coefsX)
summary(aov_mag)
```

```
##               Df Sum Sq Mean Sq F value   Pr(>F)
## indiv         42  87.53  2.0842   2.652 2.01e-06 ***
## Residuals    231 181.54  0.7859
## ---
## Signif. codes:  0 '***' 0.001 '**' 0.01 '*' 0.05 '.' 0.1 ' ' 1
```

```r
#run ANOVA on slope
aov_slope=aov((fixed_slope-mean_fixed_slope)/sd_fixed_slope ~ indiv, data= coefsX)
summary(aov_slope)
```

```
##               Df Sum Sq Mean Sq F value   Pr(>F)
## indiv         42  78.11  1.8599   2.281 5.93e-05 ***
## Residuals    231 188.37  0.8154
## ---
## Signif. codes:  0 '***' 0.001 '**' 0.01 '*' 0.05 '.' 0.1 ' ' 1
```

Figure S2

Show relative magnitudes and decay rates of different individuals.

```r
coefsX$Infection=factor(coefsX$infection,
    levels=c('Diphtheria', 'Tetanus', 'Measles',
             'Rubella', 'Vaccinia', 'Mumps*', 'VZV*'),
    labels =c('Diphtheria', 'Tetanus', 'Measles',
              'Rubella', 'Vaccinia', 'Mumps*', 'VZV*'))

interceptAOVdata <- NULL


figS2a <- ggplot(data=coefsX,
       aes(x=reorder(indiv, (fixed_int-mean_fixed_int)/(sd_fixed_int), FUN=mean),
                       y=(fixed_int-mean_fixed_int)/(sd_fixed_int))) +
    geom_boxplot() +
    geom_point(aes(color = Infection)) +
    scale_color_manual(values=infection_colors)+
    ggtitle("A. Relative magnitudes of responses of individuals")+
    ylab('normalized log(Scaled Titer)') + xlab('Indivs') + scale_x_discrete(labels=c())

figS2b <- ggplot(data=coefsX,
       aes(x=reorder(indiv, (fixed_slope-mean_fixed_slope)/(sd_fixed_slope), FUN=mean),
                       y=(fixed_slope-mean_fixed_slope)/(sd_fixed_slope))) +
```

```
    geom_boxplot() +
    geom_point(aes(color = Infection)) +
    ggtitle("B. Relative decay rates of responses of individuals")+
    scale_color_manual(values=infection_colors)+
    ylab('normalized decay rate') + xlab('Indivs') + scale_x_discrete(labels=c())
```

Plot Supplementary Figure S2.

```
# plot figures
grid.arrange(figS2a,figS2b,ncol=1)
```

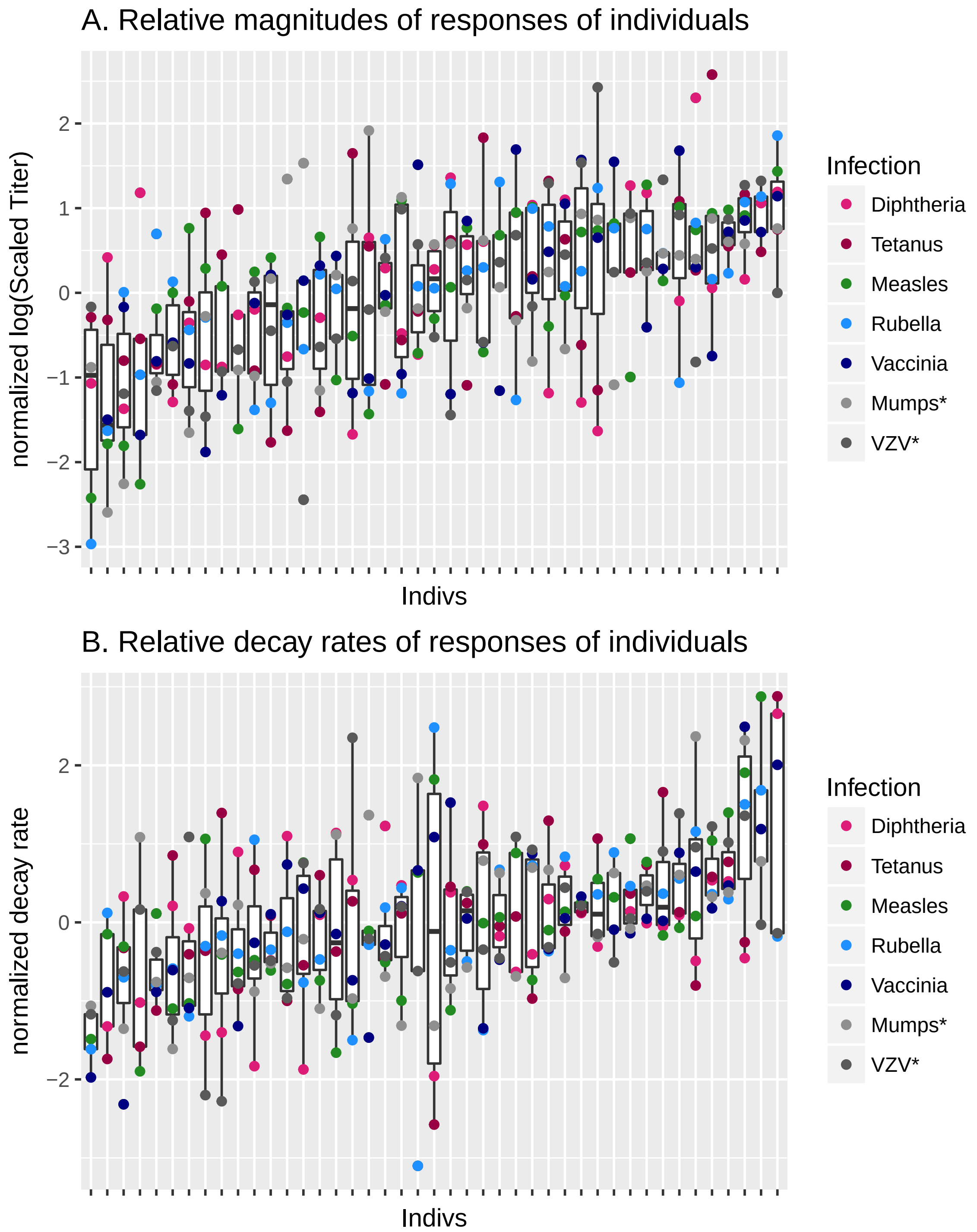


```
# export to pdf
pdf("S2_fig.pdf", height = 7, width = 12)
  grid.arrange(figS2a,figS2b,ncol=1)
dev.off()
```

```
## pdf
##   2
```
